# Oscillatory and gaze signatures of socio-emotional speech processing, visuo-spatial cognition, and their interaction in a near-realistic dual-task MEG study

**DOI:** 10.1162/IMAG.a.1134

**Published:** 2026-02-17

**Authors:** Katharina Lingelbach, Christoph S. Herrmann, Jochem W. Rieger

**Affiliations:** Applied Neurocognitive Psychology, Department of Psychology, Carl von Ossietzky Universität Oldenburg, Oldenburg, Germany; Applied Neurocognitive Systems, Fraunhofer Institute for Industrial Engineering IAO, Stuttgart, Germany; Experimental Psychology Lab, Department of Psychology, Carl von Ossietzky Universität Oldenburg, Oldenburg, Germany; Cluster for Excellence “Hearing for All”, Carl von Ossietzky Universität Oldenburg, Oldenburg, Germany; Research Center Neurosensory Science, Carl von Ossietzky Universität Oldenburg, Oldenburg, Germany

**Keywords:** magnetoencephalography (MEG), neural oscillation, pupillometry, visuo-spatial workload, socio-emotional speech processing, simulated driving, multivariate pattern analysis (MVPA)

## Abstract

In everyday settings, information processing depends on attentional focus, available cognitive resources, and stimulus characteristics such as its valence. Despite insights from laboratory studies, multisensory information processing in complex, realistic environments remains poorly understood. Using whole-head magnetoencephalography and eye tracking in a driving simulation, we addressed two research questions: (1) What are the distinct signatures of (socio-)emotional speech processing and visuo-spatial cognition in the dual task of driving and listening and (2) is information processing co-modulated by valence of speech and workload? We hypothesised two antagonistic processing modes: A top-down guided mode for (socio-)emotional speech processing and a bottom-up stimulus-driven mode for visuo-spatial cognition under high workload. A third mode was proposed to regulate emotional interference during driving under high visuo-spatial load and limited cognitive capacity. Its involvement is hypothesised to be co-modulated by valence and workload. Spatial clustering of oscillatory source activity supported the hypotheses: During emotional compared with neutral speech, parietal beta-band power increased, which likely supported processes related to predictive listening and socio-emotional cognition. In this top-down guided mode, driving-related activity decreased, as indicated by greater parietal alpha- and reduced gamma-band power. During high visuo-spatial workload, linked to the bottom-up stimulus-driven mode, gamma-band power increased in motor and orbitofrontal cortices, whereas beta-band power decreased in motor and temporo-parietal regions. Moreover, increased pupil dilation and decreased gaze dispersion were associated with this bottom-up stimulus-driven mode and visuo-spatial cognitive demands. Multivariate pattern analyses identified the third regulatory mode reflected in fronto-temporal gamma-band oscillations. It was co-modulated by valence and workload: Under low workload drives, gamma-band activity increased for negative compared with positive speech, pointing to the recruitment of inhibitory control processes. Under high workload drives with heightened visuo-spatial demands, gamma-band activity increased for positive speech, but decreased for negative speech, suggesting reduced cognitive resources and impaired control. To conclude, we found converging evidence of distinct signatures for (socio-)emotional speech, visuo-spatial cognition, and interference regulation in naturalistic multisensory environments. We propose that the top-down guided mode represents anticipatory listening and socio-emotional cognition, whereas the bottom-up stimulus-driven mode reflects the allocation of resources to driving-related spatial and sensorimotor processing but also cognitive strain under heightened task demands. Fronto-temporal gamma oscillations likely enable flexible up- and down-regulation of emotional speech processing in response to potential interference in complex, naturalistic environments.

## 1 Introduction

Our daily lives require us to constantly attend to, select, and process relevant information from multiple sensory streams, such as listening to an audiobook while monitoring the visual environment and navigating through it during driving.

### 1.1 Top-down guided and bottom-up stimulus-driven processing modes through oscillations

Oscillatory brain activity plays a crucial role in coordinating neural information processing during cognitive processes (Engel et al., 2001; Fries, 2005), attention (Clayton et al., 2015), and working memory (Engel & Fries, 2010; Wang, 2010), as well as emotional processing (Briesemeister et al., 2013). Oscillatory modulations in different frequency bands appear to be linked to distinct processing modes; for instance, whether the information is processed externally in a bottom-up stimulus-driven mode (e.g., detecting a novel, behaviourally relevant visual stimulus such as the brake lights of the car ahead) or internally in a top-down mode (e.g., reflecting on others’ emotions and intentions during a conversation; Corbetta & Shulman, 2002; Engel & Fries, 2010; Engel et al., 2001; Fries, 2005). The interplay of these two modes is proposed to facilitate goal-directed stimulus–response selection while maintaining alertness to salient novel stimuli in dynamically changing environments (Corbetta & Shulman, 2002; Fries, 2005; Huang & Elhilali, 2020); however, the extent to which each mode is engaged may differ across tasks and scenarios (e.g., Spreng et al., 2010). Gamma oscillations (>30 Hz) are proposed to be involved in bottom-up sensory processing (Bastos et al., 2015; Ou & Law, 2019), selective attention to relevant stimuli (Jensen et al., 2007; see Herrmann et al., 2010, for review), and regulatory inhibition (Kang et al., 2014; Popov et al., 2012). Specifically, gamma-band activity in the orbitofrontal cortex (OFC) appears to mediate top-down regulatory control and emotion response inhibition by communicating the context-dependent value of a stimulus and decision to the medial prefrontal cortex (mPFC; Kang et al., 2014; Popov et al., 2012; Quirk & Beer, 2006). Oscillatory modulations in the alpha frequency band (8–12 Hz) regulate sensory processing by an inhibition-based gating mechanism of stimulus-induced gamma oscillations and cortical engagement (Herring et al., 2019; Klimesch et al., 2007). Beta oscillations have been linked to top-down information processing in the auditory cortex (Arnal & Giraud, 2012; Fontolan et al., 2014; Ou & Law, 2019). Moreover, Engel and Fries (2010) proposed that beta oscillations serve as a mechanism for maintaining the “status quo”: increased beta oscillations facilitate and stabilise ongoing cognitive and motor processes, whereas unpredicted stimuli reduce beta-band oscillatory power (see also van Pelt et al., 2016).

### 1.2 Multisensory information processing in naturalistic environments

However, few neurophysiological studies have investigated information processing modes in response to complex, ecologically valid stimuli (Lingelbach et al., 2024; Lingelbach et al., 2023). As a result, the extent to which insights from strictly controlled artificial stimuli and paradigms generalise to real-world situations remains unclear. Notably, neurophysiological responses to dynamic, naturalistic emotional stimuli seem to differ from those evoked by static stimuli (Goldberg et al., 2014; Hamilton & Huth, 2020; Romeo et al., 2022).

The concurrent task of driving while listening to speech represents a safety-critical naturalistic scenario of multisensory information processing and frequent attentional shifts (see Haghani et al., 2021; Palmiero et al., 2019, for reviews).

Driving involves the continuous tracking of other agents and monitoring of the environment to detect behaviourally salient changes, including lane switching or braking vehicles, traffic signals, and road geometry such as reduced lane width (Haghani et al., 2021; Scheunemann et al., 2019). These processes engage both top-down guided and bottom-up stimulus-driven processing modes (e.g., anticipatory inference vs. visuo-spatial tracking of environmental dynamics). However, as traffic complexity rises and attentional resources are increasingly devoted to maintaining driving performance, bottom-up sensory processing becomes more prominent, prioritising immediate perceptual input to enable rapid adaptive behavioural responses (Palmiero et al., 2019).

A dual-task scenario involving concurrent speech listening has been shown to reduce activity in brain regions associated with driving-related sensory and visuo-spatial processing, as well as decrease driving performance, while simultaneously recruiting areas involved in speech processing (Fort et al., 2010; Sasai et al., 2016; Sonnleitner et al., 2012). Parietal regions linked to spatial processing and cognition, along with bilateral occipital areas, have been associated with driving (Navarro et al., 2018; Sasai et al., 2016; Spiers & Maguire, 2007), whereas temporal regions and the inferior frontal gyrus are implicated in speech processing (Friederici, 2012). It is important to note that these functional distinctions are not mutually exclusive; for instance, parietal regions can also contribute to deliberate speech processing (Grandjean, 2021; Schirmer & Kotz, 2006), and temporal regions likewise contribute to the processing of traffic-related auditory cues (e.g., Sasai et al., 2016).

In their behavioural study, Nowosielski et al. (2018) observed improved reaction times to hazards when participants listened to audiobooks during easy drives, but not during challenging ones. This finding underscores the need to examine how secondary listening tasks interact with different levels of driving-induced visuo-spatial workload.

#### 1.2.1 Socio-emotional speech processing during driving

Speech conveys meaning, intention, and emotion through both semantic and paralinguistic components. The semantic component refers to *what* is said, while the paralinguistic component relates to *how* it is expressed. The latter encompasses prosody, that is, the melodic aspect of speech (pitch, duration, and intensity; Belyk & Brown, 2014).

Emotional speech processing is proposed to operate via two neural pathways (Phillips et al., 2003; Schirmer & Kotz, 2006; Tucker et al., 1995; Wildgruber et al., 2006): One pathway operates automatically, primarily involving the superior temporal gyrus (STG) and sulcus (STS). The other relies on deliberate, controlled evaluation, engaging regions such as the right inferior frontal gyrus (IFG), OFC, dorsolateral prefrontal cortex (dlPFC), and parietal areas (see Belyk & Brown, 2014; and Witteman et al., 2012, for meta-analyses; see Grandjean, 2021, for review).

During speech perception, beta and gamma oscillations are implicated in the top-down and bottom-up information flow between hierarchical cortical levels (Arnal & Giraud, 2012; Fontolan et al., 2014). While gamma activity has been linked to forward propagation of sensory information, beta oscillations are suggested to support both the top-down transmission of content-specific predictions and rhythmic modulation of sensory sampling (Al-Zubaidi et al., 2025; Arnal & Giraud, 2012; Fontolan et al., 2014). Computational modelling approaches to speech processing further indicated that the precision of participants’ predictions is linked to oscillations in the beta-band range (Hovsepyan et al., 2023). Using intracranial recordings from the primary auditory cortex, Sedley et al. (2016) showed that oscillatory beta modulations were temporally and quantitatively aligned with the updating of sensory predictions. The authors proposed that beta activity acts as a control mechanism that gates information flow and rhythmically modulates the influence of bottom-up evidence on current internal expectations (Sedley et al., 2016). Modulations in beta-band power have also been linked to individual differences in the recruitment of top-down mechanisms (e.g., providing a categorical prediction; Ou & Law, 2019), stability and precision of phonological predictions during vowel processing (Bidelman, 2015; Scharinger et al., 2016), speech comprehensibility (Pefkou et al., 2017), and reactivation of content representations (Spitzer & Haegens, 2017; Zioga et al., 2023).

Top-down gating mechanisms modulating sensory speech processing may be particularly relevant during socio-emotional interactions to facilitate the interpretation of others’ intentions (Grandjean, 2021). In line with this notion, emotional compared with neutral prosody and socio-emotional speech content elicit increased activity in regions linked to mentalising and Theory of Mind (ToM; Grandjean, 2021). Cognitive ToM refers to the ability to represent one’s own and infer others’ thoughts, intentions, and desires (Schurz et al., 2021). These inferential and evaluative processes have been associated with temporo-parietal activity in the precuneus, cuneus, and the middle and superior temporal gyri (Schlaffke et al., 2015; Schurz et al., 2021), as well as the right temporo-parietal junction (TPJ; Saxe & Kanwisher, 2003; Schurz et al., 2021), and activation in the mPFC (Van Overwalle, 2009). Theta-, gamma-, and particularly beta-band oscillations are proposed to play a key role in coordinating information flow within and across these cortical and paralimbic regions (Mossad et al., 2022). Taken together, listening to socio-emotional speech compared with neutral speech likely enhances top-down guided modulations of the sensory input to facilitate predictive listening, reactivation of content representations, as well as inferential and evaluative processes (e.g., regarding communicative intent, emotional meaning, and social relevance).

Although the impact of conversations on driving has been widely studied at the behavioural level, and to a lesser extent, electrophysiologically (Haghani et al., 2021; Palmiero et al., 2019), very little research has investigated how socio-emotional speech with positive and negative valence impacts attention and neural information processing during driving. Assessing gaze behaviour during driving with fear-related conversations, Briggs et al. (2011) reported decreased driving performance, fewer fixations, and the phenomenon of visual tunnelling. Visual tunnelling refers to reduced gaze dispersion (i.e., increased fixation density in focal regions and decreased monitoring of the periphery) and is recognised as a marker of increased cognitive workload during driving (Nunes & Recarte, 2002).

### 1.3 Co-modulation effects of emotional speech and visuo-spatial workload

A growing body of research is investigating how cognitive and emotional processes interact when cognitive resources are limited (Brockhoff et al., 2022; Cromheeke & Mueller, 2014; Dolcos et al., 2011; Schweizer et al., 2019).

Previous studies indicate that the availability of cognitive resources (i.e., attentional control and the capacity to process information; Baddeley, 1992; Cowan, 2017; Wickens, 2014) influences how emotional stimuli are processed (Brockhoff et al., 2022; Dong et al., 2024; Tavares et al., 2016). Dual-task studies indicate that emotional processing, and thus valence-specific processing, only occurs when cognitive workload is low (Dong et al., 2024; Lingelbach & Rieger, 2025; Tavares et al., 2016). When cognitive workload is high and cognitive resources are scarce, attentional control promotes goal-directed behaviour by directing perception, processing, and response selection (Cromheeke & Mueller, 2014; E. K. Miller & Cohen, 2001). In these circumstances, the processing of task-irrelevant emotional content appears to be reduced (Brockhoff et al., 2022; Dong et al., 2024; Lingelbach & Rieger, 2025; Tavares et al., 2016). This phenomenon was evident even in cross-modal studies employing a visual task alongside auditory emotional stimuli (Mothes-Lasch et al., 2012). Mothes-Lasch et al. (2012) observed valence-specific processing for negative words during low, but not high, perceptual load in an event-related study. However, a functional near-infrared spectroscopy (fNIRS) study by Lingelbach et al. (2023) showed that participants attempted to inhibit naturalistic negative speech irrespective of the current workload level, when presented continuously in a block design. Moreover, the study reported reduced brain activity in the left temporal pole with increasing workload, suggesting a decline in the efficiency of inhibitory control (Lingelbach et al., 2023).

Several prefrontal areas play a key role in goal-directed attentional control to regulate the effects of distracting emotional information. These areas comprise the dlPFC, ventrolateral (vlPFC), and mPFC, anterior cingulate cortex (ACC), OFC, temporal pole, and IFG (Kang et al., 2014; Lingelbach & Rieger, 2025; Lingelbach et al., 2023; Ochsner et al., 2012; Popov et al., 2012).

#### 1.3.1 Gaze behaviour during multisensory information processing

Gaze behaviour is widely studied as an indicator of information processing and cognitive capacity in multisensory environments of varying complexity.

Faure et al. (2016) examined the relationship between blink activity and dual-tasking during driving. They found that blink frequency declined with increasing driving complexity. However, a secondary, auditorily presented task increased blink frequency. To date, no study has investigated the effects of socio-emotional speech or the co-modulation of socio-emotional speech and visuo-spatial complexity on blink behaviour.

Two pupillary indices, the change of pupil diameter (i.e., pupil dilation) and discontinuities in its rate of change (so-called Index of Cognitive or Pupillary Activity; hereafter IPA; see Duchowski, 2018), appear to be tied to different processing modes during dual tasking (Vogels et al., 2018). Vogels et al. (2018) found that discontinuities in pupil size changes decreased when cognitive resources are distributed across multiple tasks (i.e., in dual tasks), whereas pupil dilation increased. While the latter finding aligns with the well-established relationship between cognitive workload and pupil dilation (Beatty, 1982), the former is unexpected and contradicts previous results in pupillometry (Czerniak et al., 2021; Duchowski, 2018). Surprisingly, discontinuities increased significantly with linguistic complexity but not driving complexity (Vogels et al., 2018; see also Demberg & Sayeed, 2016).

While the relationship between pupil dilation and neural markers of attention and visuo-spatial processing is well established (Alnæs et al., 2014; Joshi et al., 2016; Vinck et al., 2015), the link between pupillary discontinuities and neural activity remains unexplored. Furthermore, their role in attention and information processing is still unclear in dual-task contexts (Demberg & Sayeed, 2016; Vogels et al., 2018).

### 1.4 Research question and hypotheses

To date, no study has integrated neural oscillatory and pupillary change discontinuities (i.e., the IPA) to investigate attentional allocation and information processing modes. Furthermore, information processing during socio-emotional speech and visuo-spatial workload under ecologically valid conditions, as well as potential interaction effects between these factors, remains unexplored, despite their relevance for driving safety. In their Road Safety Policy Framework, the European Commission identified distracted driving as a leading cause of road crashes, surpassing speed and alcohol. They emphasised the need for further research to understand and identify safety-critical mental states to develop effective warning technology (European Commission & Directorate General for Mobility and Transport, 2020). This strongly motivates the present foundational magnetoencephalography (MEG) study. Insights into the neurophysiological interplay of cognitive workload, resource capacity, and inference regulation in individual drivers can inform the design of personalised assistive systems that support safe and effective driving (Chavarriaga et al., 2018; Scheunemann et al., 2019).

Thus, this study combined whole-head MEG, offering high temporal and fair spatial resolution, with eye tracking to address two outstanding questions:

1. R.1. What are the distinct signatures of socio-emotional speech processing and visuo-spatial cognition in the dual task of driving and listening?
2. R.2. Do the valence of socio-emotional speech and workload associated with the visuo-spatial cognition co-modulate information processing?

We hypothesised two distinct, antagonistic information processing modes:

- H.1. Emotional (low, LV, and high, HV, valence) compared with neutral speech (neutral valence, NV) is expected to increase oscillatory beta-band power in parietal regions (predictive listening; Mossad et al., 2022; Sedley et al., 2016), and the number of discontinuities in pupil size changes (IPA; Vogels et al., 2018). Concurrently, oscillatory gamma-band power is expected to decrease in parietal areas linked to spatial processing (Fort et al., 2010; Sonnleitner et al., 2012).
- H.2. High compared with low visuo-spatial workload is expected to increase gamma- and decrease alpha-band power in occipital, parietal, and motor areas as a response to greater task demands. We hypothesise that the increased workload detrimentally affects driving performance. Concurrently, parietal beta-band power associated with predictive speech tracking and internalised processing is expected to decrease (Engel & Fries, 2010; Ou & Law, 2019; Sakihara et al., 2014; Sonnleitner et al., 2012). For gaze behaviour, we hypothesised increased pupil dilation, as well as decreased blink activity (Faure et al., 2016), and distribution of fixations (i.e., visual tunnelling; Briggs et al., 2011).

Regarding research question 2, we expect an interaction between the valence of emotional speech and workload (Brockhoff et al., 2022; Lingelbach & Rieger, 2025; Schweizer et al., 2019):

- H.3. Valence-specific processing and appraisal of positive speech should occur only under low workload, when cognitive resources are available. Under low workload, positive compared with negative speech is assumed to lead to higher ratings in valence and lower ratings in arousal, frustration, effort, distraction, and IPA scores. Under high workload, frontal control mechanisms in the form of gamma oscillatory activity in the mPFC and OFC are expected to regulate the processing and suppress appraisal of positive speech (Kang et al., 2014; Ochsner et al., 2012; Popov et al., 2012; Quirk & Beer, 2006). This response resembles the regulation of negative speech, which is assumed to occur irrespective of workload, to reduce emotional interference (Lingelbach et al., 2023).

## 2 Methods

### 2.1 Participants

A total of 48 volunteers participated in the study ($M_{age}=25.25$ , SD=4.01 , range = 19–38 years, 25 females and 23 males). Before the study, participants were screened to ensure they met the pre-defined inclusion criteria. The criteria included having a valid driver’s license, normal or corrected-to-normal vision, no neurological or psychiatric disorders, no history of psychoactive substance use in the last 14 days, magnetic resonance imaging (MRI) and MEG compatibility, and being a native German speaker. Participants had on average 7.9 years of driving experience (SD=3.9 , range = 1–20 years). They provided written informed consent before the experiment and received monetary compensation, with a bonus for sound driving behaviour and timely arrival at their destination. The experiment was approved by the Commission for Research Impact Assessment and Ethics at the University of Oldenburg, Germany (Ref: EK/2018/070), and conducted following the Declaration of Helsinki. For the MEG source space analysis, we excluded seven participants (N=41 , $M_{age}=25.44$ , SD=4.20 , range = 19–38 years, 20 females and 21 males) due to a missing individual MRI scan caused by a panic attack in the scanner (n=1 ), and excessive movements during driving (n=6 ; number of epochs per condition left after movement artefact rejection <30). For the eye-tracking analysis, five participants were excluded due to difficulties in obtaining a stable eye-tracking signal (N=43 , $M_{age}=25.35$ , SD=3.97 , range: 19 to 38 years, 24 females and 19 males).

### 2.2 Procedure and material

The experiment was controlled using Python 3.7. The driving scenarios were simulated with the software SILAB 6.5 (Krueger et al., 2005). The software communicated with a local Python TCP/IP socket using a binary protocol to transmit experimental triggers. We used a parallel port and Expyriment (version 0.10.0) to send synchronisation and response triggers. The triggers were stored with the MEG recordings.

#### 2.2.1 Driving simulation

The driving stimulation task was displayed using rear projection on a screen measuring 750 × 428 mm inside the magnetically shielded room of the MEG with a resolution of 1920 × 1080 pixels (PROPixx DLP LED Projector, 480 Hz refresh rate and VPixx controller). The back-projection screen was positioned 115 cm away from the participant’s eyes. The screen covered 15.07 ^°^ of visual angle in width and 8.73 ^°^ in height from the centre. The eye tracker was positioned 103 cm away from the participant’s eyes at an angle of 22.5 ^°^.

At the beginning of the experiment, participants underwent a 3-minute resting-state recording with their eyes open and fixated on a fixation cross presented centrally on a grey background. They were then given 10 minutes to familiarise themselves with the driving simulator and equipment in a practice drive.

**Fig. 1.**
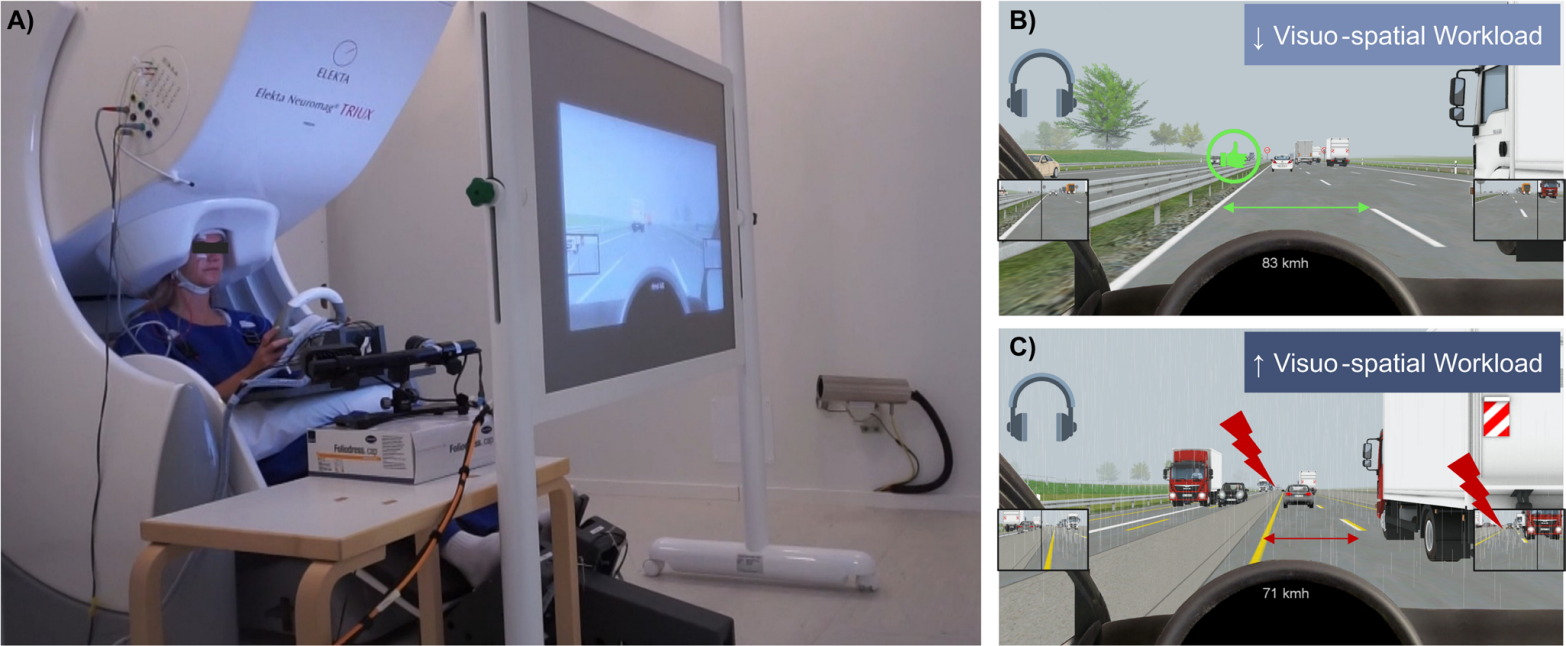
(A) Experimental set-up of the driving simulator and standard vehicle mock-up with a steering wheel, throttle, and brake pedal. (B) Low visuo-spatial workload (LW) driving sequence. (C) High visuo-spatial workload (HW) driving sequence. *Note*. Participants were seated in the MEG inside the magnetically shielded chamber.

The participants controlled the ego car in the driving simulation via a standard interface consisting of a vehicle mock-up with a throttle, brake pedal, and steering wheel (Fig. 1A). To minimise movement-related artefacts, participants were instructed to use only small, controlled movements when steering. Furthermore, they had to position their hands, arms, and elbows within a large circular support pillow that stabilised the upper body and limited excessive muscle activity. The pedals were adjusted so that only minimal foot pressure was required for braking and acceleration. During the practice trial, we monitored participants’ driving behaviour and provided feedback to help them adopt a movement-reduced driving style.

The experimental task involved six driving blocks of 12 km each, plus a 0.75 km baseline at the beginning and a 0.75 km transfer section at the end of each block. The speed limit of the baseline and transfer sections was set at 50 km/h. In other sections, the speed limit changed seven to eight times per block (mean: 70 km/h; range: 50–90 km/h). Radar controls, indicated by a red flash, and speed warnings ensured that participants did not drive too fast. Speed warnings were displayed on the windscreen whenever participants exceeded 10% of the speed limit for more than 5 seconds. Collisions with other cars triggered a yellow flash as feedback. Participants were instructed to drive as fast as possible to their destination without violating traffic rules. For violations (radar flash or collision), a penalty was deducted from the monetary bonus.

Each block represented one of the six conditions in a 2 × 3 factorial design. The two factors were (1) *visuo-spatial workload* during driving (low vs. high) and (2) *emotional auditory speech* from one or two speakers with low, high, or neutral valence. Workload was manipulated via visuo-spatial attention and driving difficulty. Low visuo-spatial workload (LW) sequences featured no construction areas (lane width = 3.75 m), three lanes, slightly foggy, dry weather, and predictable other car agents (Fig. 1B). High visuo-spatial workload (HW) sequences included construction areas with two narrower lanes (lane width = 2.75 m), slightly foggy, rainy weather, and less predictable agents exhibiting risky driving behaviours (e.g., sudden braking and tailgating; Fig. 1C). Traffic density was comparable across conditions, with six to eight vehicles randomly distributed within a 1,000 m radius of the ego car (similar to Scheunemann et al., 2019). The ego car was a typical passenger car, measuring 4.2 m in length and 1.8 m in width.

Visuo-spatial workload conditions alternated across blocks and were counterbalanced across participants. Valence of speech was randomised, ensuring no consecutive repetitions of the same valence. A representative video for each condition is provided in the Open Science Framework (OSF) project repository (https://osf.io/um6vw/).

#### 2.2.2 Auditory stimulation

Emotional naturalistic speech sequences were taken from the validated GAUDIE database (German AUDItory Emotional Database; Lingelbach et al., 2024). We included 11 stimuli from each condition in the study (metrics of the stimulus set are given in Supplementary Material Section 1). Negative speech stimuli comprised television excerpts featuring arguments between couples or relatives; positive stimuli consisted of excerpts and dialogues from comedy shows and audiobooks; and neutral stimuli comprised weather forecasts without a socio-emotional component. The audio sequences were digital-to-analog converted at a sampling frequency of 44.1 kHz using the external soundcard Fireface UCX (RME) along with an HB7 headphone amplifier (Tucker-Davis Technologies Inc., Progress Corporate Park, Alachua, Florida). The output was dichotically presented via ER3-14A insert earphones (Etymotic Research Inc.), equipped with single-use foam tips.

#### 2.2.3 Questionnaires and ratings

At the end of each driving block, participants rated their perceived effort and frustration using a modified subscale from the NASA Task Load Index (TLX, with a continuous scale ranging from 0 to 20; Hart & Staveland, 1988). They also assessed their overall valence and arousal, as well as the perceived valence and arousal of the speech, using the Self-Assessment Manikin (SAM) subscales (continuous scale ranging from 0 to 1; modified from Bradley & Lang, 1994). Additionally, participants estimated the perceived amount of distraction from the auditory speech (continuous scale ranging from 0 to 20), and answered control questions regarding the conversational content of the speech (answer option: *True*, *False*, or *Not Perceived or Memorised*).

### 2.3 Data acquisition

#### 2.3.1 Magnetoencephalogram

Neuromagnetic signals were recorded using a 306-channel whole-head MEG system (Elekta Neuromag Triux, Elekta Oy, Helsinki, Finland) with 102 magnetometers and 204 orthogonal planar gradiometers. The MEG system was housed in a magnetically shielded chamber (Vacuumschmelze, Hanau, Germany). The dewar was positioned at 68 ^°^, and participants sat upright beneath the MEG sensors. For participants with smaller head sizes, extra foam padding was used to enhance head stabilisation and limit movement. Five head position indicator (HPI) coils were attached to the participants’ heads for continuous head position tracking. To co-register the MEG signals with the structural T1 MRI scans, coil positions and anatomical landmarks (fiducials: nasion, left pre-auricular (LPA), and right pre-auricular (RPA) points) were digitised, along with at least 200 head-shape samples (Whalen et al., 2008), using the Polhemus Fastrak^®^ (Polhemus, Colchester, VT, USA). The MEG signals were recorded without internal active shielding, at a sampling rate of 1 kHz, and with an online band-pass filtering between 0.1 and 330 Hz.

#### 2.3.2 Magnetic resonance imaging (MRI) structural scans

Two structural T1-weighted MRI scans were obtained from each participant using a Siemens Magnetom Prisma 3.0 Tesla MRI machine (Siemens, Erlangen, Germany) with a 3D T1-weighted sequence (MPRAGE, TR = 2,000 ms, TE = 2.07 ms, flip angle = 9^°^, voxel size = 0.75 × 0.75 × $0.75{\text{ mm}}^{3}$, GRAPPA = 2, field of view = 240 × 240 mm, 224 sagittal slices, fat-saturated, TA = 7:45 minutes). The two T1 images were averaged to improve the signal-to-noise ratio (SNR), segmented into specific brain tissues, and individual brain surfaces were reconstructed for source localisation using FreeSurfer (version 6.0.0; Dale et al., 1999; Fischl et al., 1999).

#### 2.3.3 Eye tracking

Gaze behaviour was recorded during the experiment using the infrared remote eye-tracking device EyeLink 1000 Plus (SR Research Ltd., Ottawa, Canada) with a sampling rate of 1 kHz. At the beginning of each experiment, the eye tracker was calibrated using a 9-point calibration. At the beginning of each block, a drift correction was performed as implemented in the EyeLink software. We kept the average calibration error below 0.5 ^°^ of visual angle, with a maximum calibration error of 1.0 ^°^, and drift corrections between driving blocks below 5.0 ^°^. In cases of larger deviations, the eye tracker was recalibrated.

#### 2.3.4 Driving behaviour

Behavioural data from the simulator included the ego car’s vehicle position coordinates (X, Y, Z in m), speed (m/s), acceleration ($\text{m}/{\text{s}}^{2}$), velocity (m/s), steering wheel angle (radians/s), and the positions of the acceleration and brake pedals (continuous scale: not pressed: 0 to fully pressed: 1). The recordings were conducted via SILAB.

### 2.4 Data analysis

We analysed the data using Python 3.9 and MNE-Python (version 1.6.1; Gramfort et al., 2013).

#### 2.4.1 MEG preprocessing in sensor space

For preprocessing, the neuromagnetic signal was first decomposed into spatiotemporal components of internal and external origin using the Maxwell filter (MNE-Python default settings; Taulu & Simola, 2006; Taulu et al., 2005). In this step, flat and noisy channels were identified and reconstructed, and the data were aligned to a unified coordinate system across driving blocks. Additionally, head movements were corrected by realigning the signals to the initial head position using continuous HPI coil tracking. We examined the head movement correction by calculating the HPI coil goodness of fit (scale: 0–1), the average 3D deviation from the initial head position, and deviations between consecutive samples. The mean HPI goodness of fit exceeded 0.99. Average deviation from the initial position was 1.76 mm (SD = 0.86 mm), with 67.05% of values below 2 mm. The mean deviation between consecutive samples was 0.13 mm (SD = 0.07 mm), with 96.67% below 0.5 mm. In the next preprocessing step, raw data were downsampled to 100 Hz and band-pass filtered using a 4*^th^*-order infinite impulse response (IIR) Butterworth filter with a low cut-off frequency set at 0.1 Hz and a high cut-off at 42 Hz. Next, data from the driving blocks were concatenated. We performed a semi-automated independent component analysis on the concatenated raw data using MNE-Python (Gramfort et al., 2013), and the extended infomax algorithm (Lee et al., 1999). Electrocardiac signals were reconstructed from the MEG signals, and the blink-related artefacts were identified using information from measured electrooculography (i.e., additional passive Ag–AgCl electrodes recording horizontal and vertical eye movements). Further contaminated components (e.g., muscle and non-physiological artefacts) were manually selected based on visual inspection of the topography, time course, and power spectral intensity (Chaumon et al., 2015; Hipp & Siegel, 2013). The number of independent components was determined to retain at least 99% of the explained variance (M=74.21 , SD=2.12 , range = 68–78). On average, 8.88 components (SD = 1.72; range = 5–13) were removed per participant before back projecting the signals into sensor space. Supplementary analyses evaluating the validity of component removal and its correspondence with muscle activity are provided in Supplementary Figure S1 and Supplementary Material Section 3. After preprocessing, the continuous signals were segmented into baseline sections of 0.75 km and experimental condition sections of 12 km, based on the trigger signal marking the start of each block. From the segments, we created epochs of non-overlapping windows with an amplitude rejection for noisy epochs exceeding a threshold of 4,000 fT for magnetometers and fT/cm for gradiometers. The number of epochs was equalised across conditions by employing a method that minimised timing discrepancies across trial lists, ensuring an identical trial count per condition.

#### 2.4.2 MEG signal reconstruction to source space

To model the head’s geometry, a boundary element model (BEM; Mosher et al., 1999) was constructed using the inner skull surface extracted via the FreeSurfer watershed tessellation algorithm (Ségonne et al., 2004). A single-shell mesh was generated from this surface and assigned a conductivity of 0.3 S/m. The source space was created as a uniformly distributed grid of dipoles across the cortical surfaces according to the MNI305 (Montreal Neurological Institute) space (Collins et al., 1994), as implemented in FreeSurfer. We used icosahedron subdivisions of 5 mm, resulting in approximately 10,242 sources per hemisphere and a source spacing of 3.1 mm (i.e., a surface area per source of $9.8{\text{ mm}}^{2}$). We co-registered the MEG data with the MRI anatomy derived from individual T1 scans using a semi-automated approach following Houck and Claus (2020). Firstly, fiducials (i.e., LPA, RPA, and nasion) were estimated in the MRI head space and translated into the participant’s MRI coordinate space. The estimated landmarks derived from a template brain (Dale et al., 1999; Gramfort et al., 2013) were visually inspected and manually subject-wise adjusted. Next, we fitted the fiducials from the MRI and MEG coordinate space by scaling, translation, and rotation (relative weight for nasion = 2; LPA and RPA = 1). To align the head shape points and MRI, the iterative closest point (ICP) algorithm was applied (20 iterations; overall weight = 1). Afterwards, head shape points with a distance larger than 5 mm to the MRI skin surface were omitted, and the ICP algorithm was repeated (20 iterations; naison weight = 10; other points’ weight = 1). The co-registration resulted in a mean distance between head shape points and MRI skin surface of M=1.59 (SD=0.28 ) mm across participants. The forward model was computed by estimating the lead-field matrix using the coregistration model, source space, and BEM solution for each participant (Hämäläinen et al., 1993). The noise-covariance matrix was estimated from a combined empty room measurement of 3 minutes in duration, performed before and following the experiment. The empty room data were preprocessed similarly to the experimental MEG data. The regularisation was applied with the Ledoit–Wolf shrinkage, wherein the parameter alpha was optimised through a cross-validated search. The rank was previously calculated from the normalised data (Gramfort et al., 2013).

#### 2.4.3 Mass-univariate permutation-based spatial clustering and multivariate pattern analysis

We investigated the two research questions with two approaches: To identify neural information processing modes during socio-emotional speech and visuo-spatial cognition (R.1; main effects), we investigated differences in modulated oscillatory power in the alpha, beta, and gamma frequency bands with dynamic imaging of coherent sources (DICS) and permutation-based spatial clustering in source space. For the second research question (R.2; interaction effects), we expected rather small effect sizes at the brain level due to individual regulation strategies (Morawetz & Basten, 2024), as well as anatomical and functional variability in activation patterns across subjects (Michalke et al., 2023). Thus, we approached R.2 by applying multivariate pattern analysis (MVPA) using common spatial pattern (CSP) combined with a linear discriminant analysis (LDA). For converging evidence, we also employed mass-univariate permutation-based spatial clustering in a supplementary analysis.

Permutation-based spatial clustering is a data-driven method used to identify significant clusters of vertices that differ in their response across experimental conditions, while controlling for multiple comparisons (Maris & Oostenveld, 2007). To detect meaningful differences between conditions, a significance threshold is defined (here: p<.05 ), and measuring positions (here: vertices) are grouped based on spatial adjacency. Condition labels are then randomly reassigned across clusters in permutations (here n=5,000 ) to generate a randomised dataset. For our 2 × 3 factorial design, an *F*-value is computed for each permutation using a repeated-measures analysis of variance (rmANOVA; one-sided). A cluster is considered significant if the sum of *F*-values in the original data exceeds the $95^{\text{th}}$ percentile (p<.05 ) of the *F*-value distribution from the randomised data (Maris & Oostenveld, 2007).

MVPA is a data-driven method that employs machine learning (ML) to integrate multiple measurement sources within a multidimensional framework while taking into account inter-individual neural variability (Haufe et al., 2014; Holdgraf et al., 2017; Marsicano et al., 2024). When using linear supervised ML models combined with inverse mapping techniques (Blankertz et al., 2008; Grosse-Wentrup & Buss, 2008; Haufe et al., 2014), decoding patterns that differentiate between conditions in the classification can be derived and physiologically interpreted (Holdgraf et al., 2017). These patterns can subsequently be localised in source space (Gramfort et al., 2013).

##### 2.4.3.1 Modulated oscillatory power with dynamic imaging of coherent sources (R.1)

To localise modulated oscillatory power of emotional speech processing and visuo-spatial cognition (R.1), we used the beamformer DICS (J. Gross et al., 2001; van Vliet et al., 2018). This frequency-domain beamformer constructs spatial filters in source space to estimate frequency-specific activity from a target location, while suppressing contributions from other sources (van Veen et al., 1997; van Vliet et al., 2018). For DICS, cross-spectral density (CSD) matrices were computed using Morlet wavelet decomposition on 5-second epochs (3 cycles, frequencies from 1–42 Hz). The number of epochs per condition was on average M=99.83 (SD=16.01 , range: 33–115; $M_{drop}=19.15\%$ ). The CSDs and the forward model were used to construct the spatial filters for each cortical grid point. These spatial filters were then applied condition-wise to calculate the relative change in each condition from its baseline across frequencies (van Vliet et al., 2018). To combine magnetometer and gradiometer signals, data were spatially pre-whitened using a noise-estimating covariance matrix from the empty room measurement (Engemann & Gramfort, 2015; Gramfort et al., 2013).

Oscillatory activity within canonical frequency bands is superimposed upon a broadband, non-oscillatory (aperiodic) background component (Donoghue et al., 2020;K. J. Miller et al., 2009). This component exhibits a scale-free, 1/*f* -like spectral profile (He et al., 2010; K. J. Miller et al., 2009). It is assumed to be evoked by the asynchronous summation of postsynaptic potentials across large neuronal populations, reflecting the underlying balance of excitatory and inhibitory synaptic activity and their respective time constants (e.g., He et al., 2010; K. J. Miller et al., 2009). In recent years, increasing attention has been directed towards the aperiodic aspects of neural power spectra (Donoghue et al., 2020; Gerster et al., 2022; Lu et al., 2024; Thuwal et al., 2021). Importantly, the 1/*f* signal appears to be also modulated by task-related experimental manipulations and may reflect not only background neural noise but also physiologically meaningful activity (Donoghue et al., 2020; Lu et al., 2024; Zhang et al., 2023). Consequently, changes in the aperiodic components can confound or even obscure modulations in oscillatory power above the 1/*f* -like activity (Donoghue et al., 2020; Gerster et al., 2022). This is particularly true for experimental paradigms that are not stimulus locked relative to a pre-stimulus baseline. These considerations motivate the separation of the aperiodic and oscillatory components above this 1/*f* -like part for independent investigation.

To separate 1/*f* -like activity from the superimposed oscillatory activity, we visually inspected the power spectra to ensure there were no apparent objections to decomposing the signal components as proposed by Gerster et al. (2022). Afterwards, we estimated the aperiodic component, including the offset and slope of the 1/f decay, using the FOOOF algorithm (version 1.1.0; Donoghue et al., 2020). This was performed for each participant, condition, and vertex with the following parameters: peak width limits = 2–6 Hz, maximum number of peaks = 7, absolute peak detection threshold = 0.0, relative peak detection threshold = 1.0, and a fixed mode. The aperiodic component was then subtracted from the power spectral density in linear space (Gyurkovics et al., 2021).

Supplementary Figure S5 shows the power spectrum separated from the 1/*f* -like signal (solid lines) and aperiodic component (dotted lines; A–C) for the single effect (A) and main effect conditions (B: visuo-spatial workload; C: emotional speech).

To obtain the power within the frequency bands of interest, individual band peaks were estimated from resting-state data in sensor space. Similar to the processing of the conditions, non-overlapping 5-second epochs were extracted from the resting-state data, and power was calculated using the multitaper method with a bandwidth of 2 and averaged across epochs for both sensor types. Peak frequencies for the alpha, beta, and gamma bands were then identified for each sensor using the FOOOF algorithm (Donoghue et al., 2020), constrained to the following cut-off ranges: 8–12 Hz (alpha), 15–25 Hz (beta), and 30–42 Hz (gamma). Peak frequencies were averaged across all sensor positions and types, and the source-space frequency band power was calculated using a 2 Hz bandwidth centred on the individual peak. The average ${\text{R}}^{2}$ of the power parametrisation models was M=0.97 (SD=0.01 ), with a mean error of M=0.03 (SD=0.01 ). Descriptive statistics of the band centre frequencies are provided in Supplementary Material Section 4 and Supplementary Table S1. The narrowband oscillatory power was averaged for each frequency band, and the spatial distribution of the individual oscillatory power of each condition and frequency band was morphed to the average FreeSurfer brain template fsaverage (Fischl et al., 1999; Gramfort et al., 2013). This morphing transforms the source space of individual subjects into a common source space, enabling group-level statistical analysis. Grand averages of the DICS localised oscillatory source power per frequency band and main effect condition (R.1) are visualised in Supplementary Figure S6 (with subtraction of the aperiodic part) and Supplementary Figure S7 (without subtraction of the aperiodic part).

For the non-parametric permutation-based clustering statistic, we tested for significant main effects of emotional speech and visuo-spatial workload using an rmANOVA (Maris & Oostenveld, 2007). Potential interaction effects were also examined to rule out cross-over interactions that could compromise the interpretability of the main effects. *F*-values of significant clusters were projected on a 3D brain (*fsaverage*). In the presence of a significant main effect, oscillatory power was averaged across the vertices of the significant *F*-test cluster. Pairwise Wilcoxon signed-rank tests (a non-parametric alternative to the dependent *t*-test; scipy version 1.5.0) were then conducted for the respective contrasts to determine which conditions differed in cluster-wise modulations. Family-wise error correction was applied using the false discovery rate (FDR) via the Benjamini–Hochberg method (statsmodels; version 0.13.5).

Exploratory analyses of changes in the aperiodic components in response to emotional speech and visuo-spatial workload are presented in Supplementary Material Section 8 and Supplementary Figure S9. Supplementary analyses of modulations in band source power that were not corrected for the aperiodic component are provided in Supplementary Material Section 9 and Supplementary Figure S10.

To investigate the relationship between significant neural markers of information processing during emotional speech and visuo-spatial workload (R.1), we conducted correlation analyses using Spearman rank correlations ($r_{s}$). Average cluster source band power was computed over the vertices of significant clusters of the main effects of emotional speech and workload. To account for multiple correlations, the significance level was set at α=0.01 .

##### 2.4.3.2 Interaction effect of valence and workload on modulated oscillatory power (R.2)

To investigate the second research question (R.2; interaction effects), we selected a shorter epoch length of 2 seconds (non-overlapping) to increase the number of epochs per condition for the ML decoding. The number of epochs was equalised across conditions with an average of M=275.12 (SD=11.54 , range: 237–296; $M_{drop}=8.17\%$ ) per condition. In the MVPA, the cleaned raw data were band-pass filtered using a linear-phase finite impulse response (FIR) filter with zero-padding in the gamma-band frequency range (cut-offs: 30 and 42 Hz) before epoching. Next, we applied MVPA to the epoched band-pass filtered sensor-space data using a multiclass extension of CSP (Blankertz et al., 2008; Grosse-Wentrup & Buss, 2008, as implemented in MNE-Python), combined with a linear discriminant analysis (LDA; implemented in scikit-learn 1.5.2). The decoding was performed using the LDA with least-squares solution as solver to discriminate the four experimental conditions in a within-subject four-class classification (emotional speech/visuo-spatial workload conditions: HV/LW, LV/LW, HV/HW, LV/HW). We selected the first four CSP components (log-transformed) as input for the LDA. Covariance matrices in both the multiclass CSP extraction and LDA were regularised using the Ledoit–Wolf method. We used the Pipeline method to streamline all preprocessing and classification steps (scikit-learn 1.5.2). Each participant’s data (shape: $n_{epochs}$ , 306 channels, 200 time points) were partitioned into epochs for training and testing using repeated stratified 10-fold cross-validation with 3 repetitions (30 folds total). To evaluate decoding performance, we used the *F*1 score and analysed confusion matrices across participants. The subject-wise mean *F*1 scores and their 95% confidence intervals (CIs) were computed by bootstrapping across cross-validation folds using a Monte Carlo simulation (MCS with 5,000 iterations; Cumming & Finch, 2005). The spatial patterns of the CSP component used in the decoding were derived from the fitted linear models and averaged across cross-validation folds. Further methodological information regarding CSP for multiclass decoding is given in Supplementary Material Section 5.

Although DICS is well suited for investigating oscillatory power modulations in source space, it estimates spatial filters using a common CSD matrix that combines information across all experimental conditions. However, creating a common CSD matrix is not applicable when transforming CSP components derived from MVPA decoding to source space. Therefore, we chose Minimum Norm Estimate (MNE) as inverse solution for source reconstruction to investigate R.2 and H.3. MNE estimates the most likely distribution of neural sources by selecting the one with the minimum overall current amplitude (as measured by the L2 norm) that explains the observed magnetic fields (Hämäläinen et al., 1993). It is particularly useful when the source configuration is complex or unknown (Hauk, 2004). For the localisation of the CSP component patterns in source space, the SNR was set at 3. To estimate gamma-band power from the inverse solution of the epoched data, we used the multitaper method with discrete prolate spheroidal sequence (DPSS) windows, a 2 Hz bandwidth, and an SNR of 1 (Gramfort et al., 2013). Gamma-band estimates were used to interpret component patterns in relation to the experimental conditions. In both cases (i.e., CSP component patterns and gamma-band oscillatory power), the depth prior was estimated from the data using a weighting exponent of 0.8. We applied a loose orientation constraint with a weighting factor of 0.2 (Lin et al., 2006), and computed the norm of the resulting loose orientations. Decoding patterns and gamma-band power estimates in source space were then aligned across participants by morphing them to the *fsaverage* template brain (Fischl et al., 1999; Gramfort et al., 2013). Grand averages were computed by weighting each participant’s activation pattern according to their test decoding *F*1 score, thereby enhancing the influence of participants with higher classification performance. Subsequently, the resulting averages were rescaled to a 0–1 range and visualised on the 3D *fsaverage* brain template.

To examine how the CSP component patterns relate to the emotional speech and visuo-spatial workload conditions, gamma-band source power was averaged across vertices whose absolute decoding values exceeded the $90^{\text{th}}$ percentile in the CSP pattern. Differences between conditions were assessed using Wilcoxon signed-rank tests with FDR correction, and visualised via bootstrapped condition means and their CIs (MCS with 5,000 iterations).

In a final supplementary analysis, we compared the MVPA results with a traditional mass-univariate, permutation-based clustering (Maris & Oostenveld, 2007). Consistent with the approach used for R.1, an rmANOVA with a one-sided omnibus *F*-test was applied to identify clusters reflecting interaction effects in gamma-band source power localised with MNE. In case of a significant interaction effect, oscillatory power was averaged across the vertices of significant clusters, and Wilcoxon signed-rank tests were performed (FDR-corrected). As before, results were visualised using bootstrapped condition means and their CIs (MCS with 5,000 iterations).

#### 2.4.4 Analysis of subjective ratings

To investigate our second research question of a co-modulation on the subjective experience (R.2, H.3), we analysed self-reported ratings collected at the end of each driving block. The effort and frustration ratings, originally collected on a 0–20 scale in the NASA-TLX, as well as the distraction scale, were rescaled to a 0–1 range to match the scale used for the SAM, and to facilitate interpretation. Recall and missed content in the conversation questions were dummy coded for further supplementary analyses.

#### 2.4.5 Analysis of driving behaviour

We used the following measures to assess driving performance: changes in (1) acceleration and (2) steering wheel angle, both computed as the root mean square of successive differences (*RMSSD*); (3) average brake actuation; (4) traffic rule violations, including the number of radar flashes and collisions; and (5) mean lane deviation from the centre line, excluding 2 seconds around each lane change. These measures were aggregated into an overall driving performance score. This was done by first normalising each measure to a 0–1 range, averaging across measures, and inverting the aggregated score so that higher values indicated better performance. The final performance score was then rescaled once more, yielding values ranging from 0 (indicating poor performance) to 1 (indicating high performance).

#### 2.4.6 Analysis of gaze-related measures

To assess gaze-related changes in information processing under varying visuo-spatial workload and emotional speech, we focused on four measures: (1) gaze dispersion, (2) blink rate per second, and two pupillary measures, (3) the average pupil dilation, and (4) IPA, reflecting the number of abrupt, sharp fluctuations in pupil diameter per second. All measures were baseline corrected by subtracting the value obtained during the baseline block from the value in the immediately following experimental block.

Blinks, including minimal, missing, or distorted pupil signals due to eyelid closure, were identified by an online event parser of the EyeLink eye tracker. The number of blinks per driving block (i.e., condition) was counted and normalised by the block duration (in seconds). We preprocessed the gaze signals by interpolating missing data (due to blinks and artefacts). Interpolation was done using a cubic spline method, with 50 ms padding around each gap and a maximum tolerated data loss of 500 ms (Kret & Sjak-Shie, 2019; Mathôt et al., 2018). The time series were then filtered using a median-based rolling window of 20 ms.

##### 2.4.6.1 Calculation of gaze dispersion

We quantified gaze dispersion using the root mean square (*RMS*) distance of fixations from the gaze centroid within each driving block (i.e., condition). The gaze centroid was defined as the duration-weighted mean of the x- and y-coordinates across all fixations. Implementation details are provided in Supplementary Material Section 6.

##### 2.4.6.2 Calculation of pupillary measures

For the pupillary measures, pupil diameter was first converted to millimetres (see Lingelbach & Rieger, 2025, details can also be found in Supplementary Material Section 6). Afterwards, we removed outliers below 1 mm and above 9 mm (outside the physiologically valid range; Kret & Sjak-Shie, 2019). Finally, the median pupil diameter during fixations was computed. Fixation events were obtained via the online event parser of the EyeLink eye tracker.

The second pupillary measure IPA quantifies the fluctuations in pupil diameter as the frequency of abrupt discontinuities detected within the signal (in events per second; Duchowski, 2018). To compute the IPA, we applied a wavelet-based algorithm described in detail by Duchowski (2018), and in Supplementary Material Section 6 and Supplementary Figure S2.

#### 2.4.7 Inferential statistics of subjective, behavioural, and gaze-related correlates

For the subjective ratings, driving performance, and gaze-related measures, we used linear mixed-effects models (LMMs; Baayen et al., 2008), as implemented in the toolbox pymer4 (version 0.8.0; Jolly, 2018), to investigate main effects of the factors emotional speech (R.1; H.1) and visuo-spatial workload (R.1; H.2), as well as their interaction (R.2; H.3). Outliers exceeding the 95*^th^* percentile were excluded and values rescaled using a z-score standardisation. Furthermore, the participant variable was incorporated as a random intercept in the models to account for non-systematic variations among individuals. Fixed effects were tested using *F*-statistics derived from Type-III sums of squares. If distributional assumptions were violated, the model was reparameterised and refitted using orthogonal polynomial contrasts before computing the ANOVA results. Degrees of freedom for fixed effects were estimated using the Satterthwaite approximation. Similar to the MEG analyses, post-hoc comparisons of significant effects were performed by using FDR-corrected Wilcoxon signed-rank tests, and visualising via bootstrapped means and their CIs (MCS with 5,000 iterations) of the contrasts (main effects; R.1 and H.1–2) and conditions (interaction effects; R.2 and H.3).

## 3 Results

### 3.1 Distinct processing modes for socio-emotional speech and visuo-spatial workload (R.1)

To address our first research question of distinct information processing modes during socio-emotional speech and visuo-spatial cognition (R.1; main effects), we applied permutation-based spatial clustering (Maris & Oostenveld, 2007) to oscillatory source space power localised with DICS (J. Gross et al., 2001) to investigate modulations in the alpha, beta, and gamma bands. Since we were interested in the main effects, we averaged the source band power across epochs of the respective other factor.

The permutation-based spatial clustering analyses revealed two distinct information processing modes: a top-down guided mode associated with socio-emotional speech and a bottom-up stimulus-driven mode associated with visuo-spatial workload, supporting H.1 and H.2.

Supplementary Figure S8 presents the mean cluster band power per condition for the significant main effects. There was no significant interaction effect. Hence, all main effects can be interpreted.

For subjective ratings, driving performance, and gaze behaviour, linear mixed-effects models were used to examine the distinct information processing modes of socio-emotional speech and visuo-spatial workload. Detailed statistics on the subjective ratings are provided in Supplementary Material Section 7, Supplementary Table S2, and Supplementary Figure S3, along with supplementary analyses on speech valence, speech arousal, and recall (Supplementary Material Sections 2 and 7, Supplementary Table S3). Descriptive statistics and a detailed statistical summary on the driving performance and gaze behaviour are given in Supplementary Figure S4A–D, Supplementary Material Section 7, and Supplementary Table S4, respectively.

#### 3.1.1 Signatures of a top-down guided processing mode for socio-emotional speech (H.1)

##### 3.1.1.1 Modulated oscillatory power with dynamic imaging of coherent sources

**Fig. 2.**
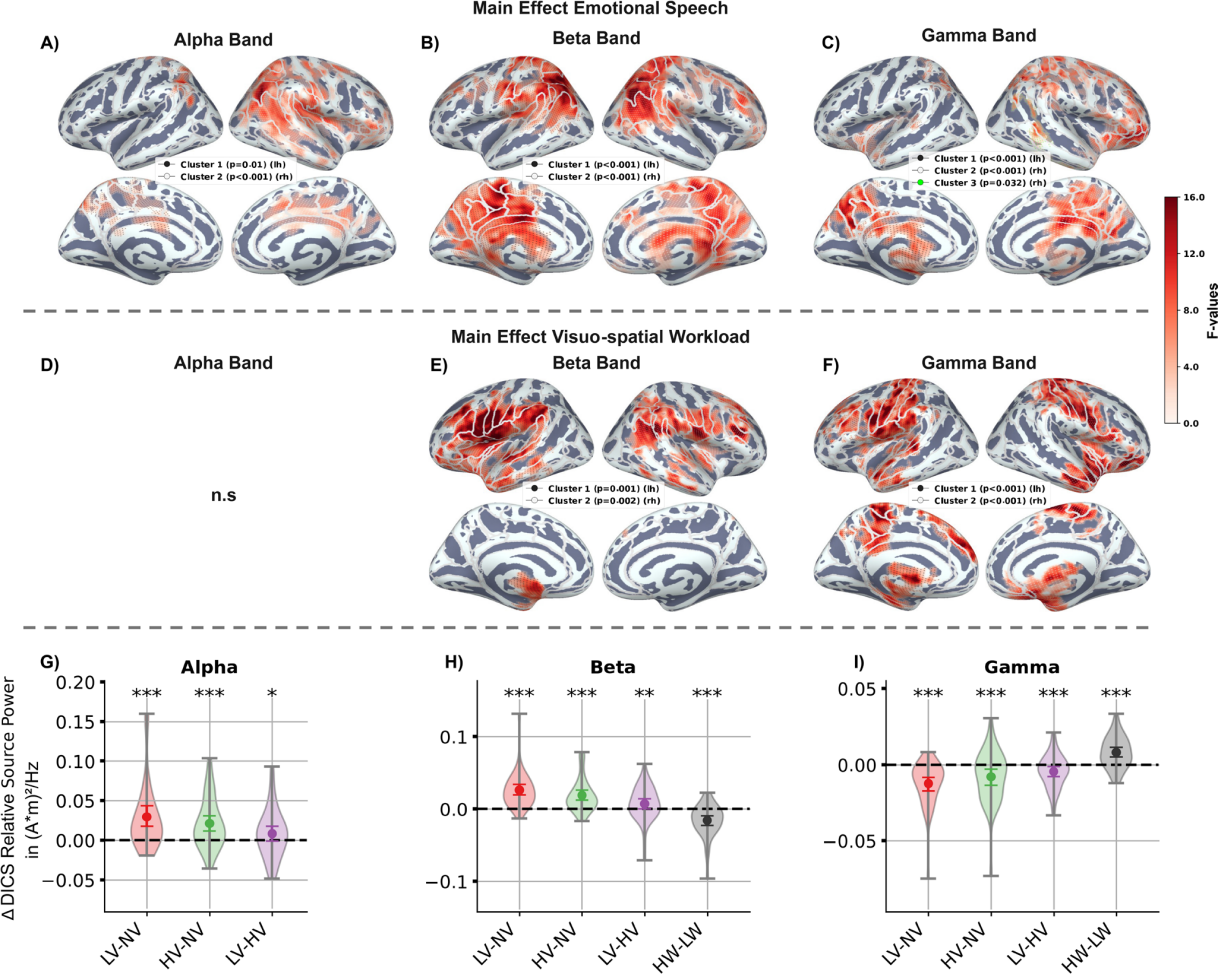
Results of the permutation-based spatial clustering of source power localised with DICS. *Note.* The spatial distribution of significant *F*-values from the *F*-test clusters for the main effect of emotional speech is shown in Panel (A) for alpha-band power, (B) for beta-band power, and (C) for gamma-band power. The spatial distribution of significant *F*-values for the main effect of visuo-spatial workload is displayed in Panel (E) for beta-band power and (F) for gamma-band power. Results and contrasts of the post-hoc Wilcoxon signed-rank tests are shown in Panels (G) to (I). Band power was averaged across vertices within significant *F*-test clusters for each frequency band and effect. The shaded area indicates the estimated probability density of the data. Significance levels from the Wilcoxon signed-rank test, corrected using false discovery rate: *** for p<.001 , ** for p<.01 , * for p<.05 . rh: right hemisphere, lh: left hemisphere. LV: low valence; NV: neutral valence; HV: high valence; LW: low visuo-spatial workload; HW: high visuo-spatial workload.

In the DICS oscillatory power analysis, we found clusters yielding a significant main effect of emotional speech on modulations in alpha (*F*-statistic; left hemisphere (lh): p=.01 ; right hemisphere (rh): p<.001 ; Fig. 2A), beta (lh and rh: p<.001 ; Fig. 2B), and gamma-band power (lh and rh: p<.001 ; additional cluster 3 in rh: p=.032 ; Fig. 2C).

The alpha-band cluster was primarily located in parietal regions implicated in spatial cognition and attention (Fort et al., 2010; Sonnleitner et al., 2012). The clusters comprised the superior parietal lobe, precuneus, right angular gyrus, and posterior cingulate cortex (Fig. 2A). Beta-band clusters encompassed bilateral superior and inferior parietal lobes, the precuneus, intraparietal sulcus, angular gyrus, and cingulate cortex (Fig. 2B). Gamma-band clusters included the bilateral precuneus and right OFC, with an additional cluster located near the posterior STS and right TPJ (Fig. 2C).

The post-hoc Wilcoxon signed-rank tests contrasting the speech conditions revealed increased alpha-band power during emotional compared with neutral speech (LV – NV: M=0.03 , 95% CI [0.02, 0.04] ; $Z_{\left(47\right)}=-6.53$ , p<.001 ; HV – NV: M=0.02 , 95% CI 0.01, 0.03] ; $Z_{\left(47\right)}=-5.17$ , p<.001 ), and a marginally significant increase for negative compared with positive speech (LV – HV: M=0.01 , 95% CI [0.00, 0.02] ; $Z_{\left(47\right)}=-2.15$ , p=.031 ; Fig. 2G).

Similarly, beta-band power increased with emotional speech (LV – NV: M=0.03 , 95% CI [0.02, 0.03] ; $Z_{\left(47\right)}=-7.62$ , p<.001 ; HV – NV: M=0.02 , 95% CI [0.01, 0.03] ; $Z_{\left(47\right)}=-6.69$ , p<.001 ; LV – HV: M=0.01 , 95% CI [0.00, 0.01]; $Z_{\left(47\right)}=-3.02$ , p=.003 ; Fig. 2H).

Conversely, gamma-band power decreased in response to emotional speech (LV – NV: M=−0.01 , 95% CI [−0.02, −0.01] ; $Z_{\left(47\right)}=-7.22$ , p<.001 ; HV – NV: M=−0.01 , 95% CI [−0.01, 0.00] ; $Z_{\left(47\right)}=-3.86$ , p<.001 ), with a stronger decrease observed for negative than for positive speech (LV – HV: M=−0.01 , 95% CI [−0.01, 0.00] ; $Z_{\left(47\right)}=-3.47$ , p<.001 ; Fig. 2I).

##### 3.1.1.2 Subjective experience during emotional speech

Linear mixed-effects models revealed main effects of emotional speech for valence, arousal, frustration, distraction, and effort. Due to significant interactions on valence, arousal, and frustration ratings, only the main effects of emotional speech in the contrast between HV and NV can be interpreted for valence ($F_{\left(2,207.76\right)}=11.34$ , p<.001 ) and frustration ($F_{\left(2,221,28\right)}=9.72$ , p<.001 ).

**Fig. 3.**
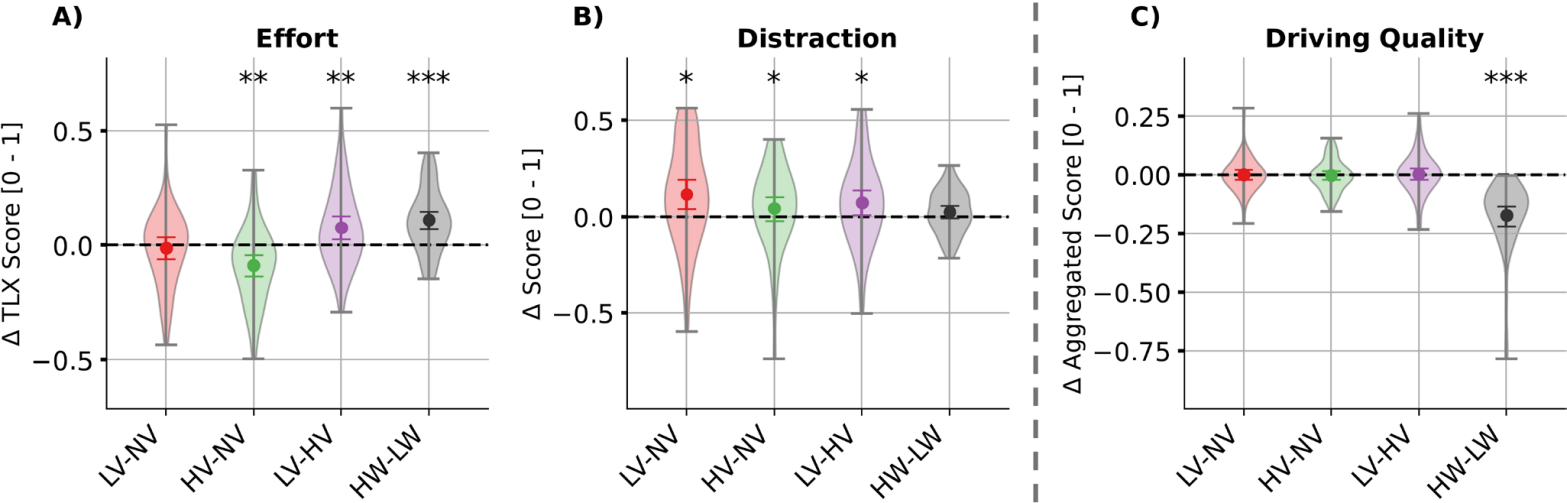
Post*-*hoc comparisons of main effects of emotional speech and visuo-spatial workload in the subjective ratings of perceived (A) effort and (B) distraction, as well as (C) driving quality. *Note.* Coloured dots and error bars represent the bootstrapped grand averages and their Bonferroni-corrected 2.5*^th^* and 97.5*^th^* confidence interval (CI) across participants. The shaded area indicates the estimated probability density of the data. LV: low valence; NV: neutral valence; HV: high valence; LW: low visuo-spatial workload; HW: high visuo-spatial workload. Significance level from the Wilcoxon signed-rank test corrected with false discovery rate: *** for p<.001 , ** for p<.01 , * for p<.05 .

The post-hoc tests indicate that drives with positive speech elicited higher valence (HV – NV: M=0.11 , 95% CI [0.07, 0.14] ; $Z_{\left(47\right)}=-4.23$ , p<.001 ) and lower frustration ratings (HV – NV: M=−0.09 , 95% CI [−0.12, −0.05] ; $Z_{\left(47\right)}=-4.24$ , p<.001 ) than those with neutral speech, regardless of the current workload. In addition, listening to positive speech was rated as less effortful compared with both negative and neutral speech (main effect of emotional speech: $F_{\left(2,206.97\right)}=4.82$ , p=.009 ; 11.63% outliers excluded; post-hoc tests: LV – HV: M=0.08 , 95% CI [0.03, 0.13] ; $Z_{\left(47\right)}=-275$ , p=.009 ; HV – NV: M=−0.09 , 95% CI [−0.13, −0.04] ; $Z_{\left(47\right)}=-3.50$ , p=.001 ; Fig. 3A; H.1).

Concerning distraction ratings, emotional speech was perceived as more distracting during the drives (distraction: $F_{\left(2,195.89\right)}=5.90$ , p=.003 ; 11.57% outliers excluded). Specifically, negative speech was rated as more distracting than both neutral (LV – NV: M=0.12 , 95% CI [0.04, 0.19] ; $Z_{\left(44\right)}=-2.88$ , p=.012 ) and positive speech (LV – HV: M=0.07 , 95% CI [0.01, 0.14] ; $Z_{\left(44\right)}=-2.22$ , p=.040 ; Fig. 3B; H.1). There was a marginal effect suggesting greater distraction from positive than from neutral speech (HV – NV: M=0.04 , 95% CI [−0.02, 0.10] ; $Z_{\left(44\right)}=-1.96$ , p=.0499 ). An exploratory analysis using Spearman rank correlations ($r_{s}$) revealed that ratings of perceived effort and distraction, as well as speech arousal, were highly correlated within participants (effort – distraction: $r_{s}=.459$ , p<.001 ; effort – speech arousal: $r_{s}=.316$ , p<.001 ; distraction – speech arousal: $r_{s}=.518$ , p<.001 ; df=46 ).

##### 3.1.1.3 Gaze behaviour

**Fig. 4.**
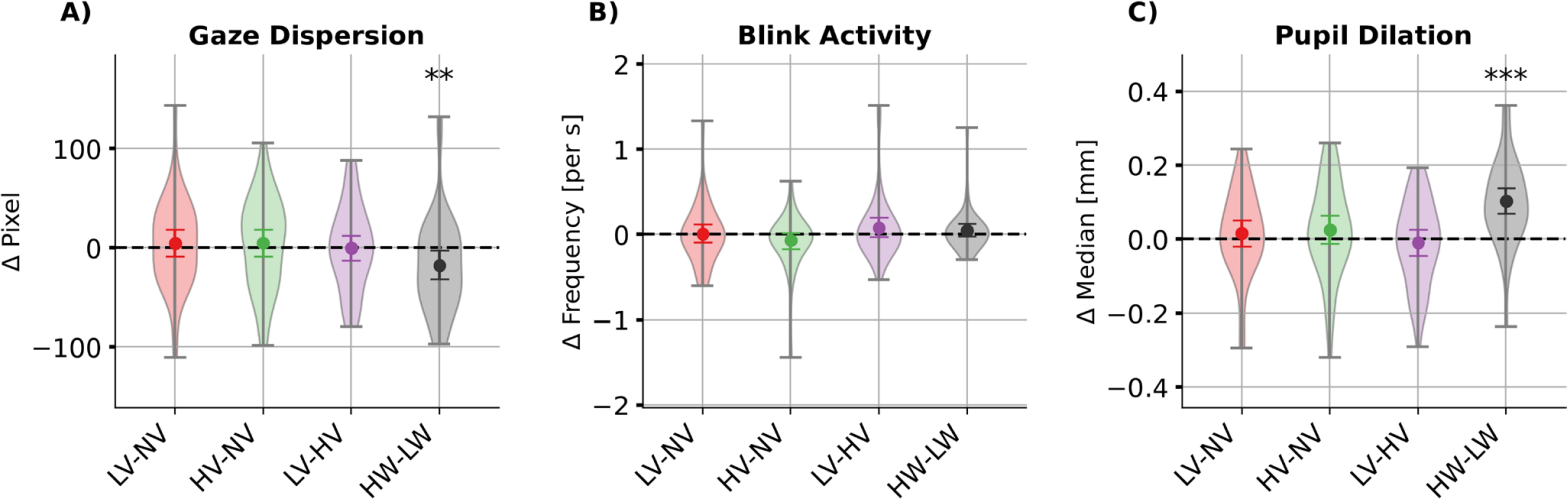
Post-hoc comparisons of main effects of emotional speech and visuo-spatial workload in the gaze behaviour measures (A) gaze dispersion, (B) blink activity, and (C) pupil dilation. *Note.* Coloured dots and error bars represent the bootstrapped grand averages and their Bonferroni-corrected 2.5*^th^* and 97.5*^th^* confidence interval (CI) across participants. The shaded area indicates the estimated probability density of the data. LV: low valence; NV: neutral valence; HV: high valence; LW: low visuo-spatial workload; HW: high visuo-spatial workload. Significance level from the Wilcoxon signed-rank test corrected with false discovery rate: *** for p<.001 , ** for p<.01 , * for p<.05 .

Regarding gaze behaviour, we did not observe a significant effect of emotional speech on the number of discontinuities in pupil size changes (IPA; contrary to H.1). Conversely, emotional speech did affect blink frequency ($F_{\left(2,180.12\right)}=4.53$ , p=.012 ; excluded outlier observations: 11.50% ) and pupil dilation ($F_{\left(2,179.35\right)}=3.95$ , p=.021 ; excluded outlier observations: 11.50% ). However, post-hoc tests did not reveal significant pairwise differences (Fig. 4B).

##### 3.1.1.4 Interim summary of the top-down guided processing mode

Taken together, we observed increased fronto-parietal beta-band power, alongside decreased fronto-parietal gamma-band and temporo-parietal alpha-band power, as distinct neural markers of socio-emotional compared with neutral speech processing. Specifically, the oscillatory modulation of beta-band power in parietal regions (i.e., the precuneus, TPJ, and inferior parietal lobe) has previously been linked to top-down guided processing, including predictive listening, as well as inferential and evaluative socio-emotional processes (e.g., Grandjean, 2021; Mossad et al., 2022).

At the subjective level, socio-emotional speech was perceived as more distracting than neutral speech. Negative speech was rated as the most distracting, while positive speech showed only a trend towards increased distraction relative to neutral speech. Moreover, positive speech was experienced as more pleasant and less frustrating than neutral speech, and as less effortful than both neutral and negative speech. Explorative correlational analyses suggest a link between speech arousal and the degree of perceived distraction and effort.

#### 3.1.2 Signatures of a bottom-up stimulus-driven processing mode for high visuo-spatial workload (H.2)

##### 3.1.2.1 Modulated oscillatory power with dynamic imaging of coherent sources

In the cluster analysis, significant clusters were identified in the beta (lh: p=.001 ; rh: p=.002 ; Fig. 2E) and gamma bands (lh and rh: p<.001 ; Fig. 2F) for the main effect of visuo-spatial workload.

The beta-band cluster encompassed frontal regions such as the pars opercularis of the IFG, inferior dlPFC, frontal operculum, and anterior insula, as well as areas above the Sylvian fissure, including the inferior precentral gyrus and TPJ (Fig. 2E). Gamma-band clusters covered the motor and parietal cortices, including the posterior cingulate cortex and precuneus, as well as the left frontal eye field, and mPFC and OFC (Fig. 2F).

Post-hoc tests showed that gamma-band power increased in high compared with low visuo-spatial load drives (HW – LW: M=0.01 , 95% CI [0.00, 0.01] ; $Z_{\left(47\right)}=-4.11$ , p<.001 ; Fig. 2I), while beta-band power decreased (HW – LW: M=−0.02 , 95% CI [−0.02, −0.01] ; $Z_{\left(47\right)}=-4.14$ , p<.001 ; Fig. 2H).

##### 3.1.2.2 Subjective experience during increased visuo-spatial workload

A significant main effect of workload on perceived effort ($F_{\left(1,207.24\right)}=18.42$ , p<.001 ; 11.63% outliers excluded) indicated that participants rated high visuo-spatial workload as more effortful than low workload (HW – LW: M=0.11 , 95% CI [0.07, 0.15] ; post-hoc test: $Z_{\left(47\right)}=-4.48$ , p<.001 ).

##### 3.1.2.3 Driving performance

In line with the subjective ratings, results of the linear mixed-effects model for driving quality confirmed that it was more difficult to navigate through driving scenarios with high compared with low visuo-spatial workload (manipulation check). This was reflected in a significant main effect of visuo-spatial workload ($F_{\left(1,215.53\right)}=263.22$ , p<.001 ; excluded outlier observations: 11.63% ), and significantly lower driving quality under high compared with low workload (HW – LW: M=−0.17 , 95% CI [−0.22, −0.13] ; $Z_{\left(47\right)}=-6.03$ , p<.001 ; Fig. 3C). We observed significant main effects of visuo-spatial workload in all subvariables of the driving quality score (i.e., acceleration *RMSSD*, steering wheel angle *RMSSD*, mean brake actuation, mean deviation from lane centre, and number of traffic rule violations), supporting our decision to aggregate them into one score (see Supplementary Table S4 and its note for details). There were no differences between the emotional speech conditions, nor was there a significant interaction effect.

##### 3.1.2.4 Gaze behaviour

Next, we investigated whether high visuo-spatial workload increases pupil dilation and reduces blink frequency and gaze dispersion using linear mixed-effects models (H.2).

In line with H.2, we observed a significant main effect of visuo-spatial workload on pupil dilation ($F_{\left(1,182.88\right)}=$ 37.53 , p<.001 ; excluded outlier observations: 11.50% ), with larger pupil size during high workload (HW – LW: M=0.14 , 95% CI [0.07, 0.10] ; $Z_{\left(42\right)}=-4.67$ , p<.001 ; Fig. 4C). In addition, gaze dispersion reduced ($F_{\left(1,181.51\right)}=$ 9.95 , p=.002 ; excluded outlier observations: 11.50% ), indicating visual tunnelling (HW – LW: M=−2.74 , 95% CI [−31.33, −17.57] ; $Z_{\left(42\right)}=-2.82$ , p=.004 ; Fig. 4A). Contrary to our hypothesis, blink frequency was not significantly affected by increasing visuo-spatial workload in the driving task.

##### 3.1.2.5 Interim summary of the bottom-up stimulus-driven processing mode

To summarise, high visuo-spatial workload increased task demands, as reflected by increased subjective effort and pupil dilation, as well as reduced driving quality and gaze dispersion (in line with H.2). This heightened task demand appeared to elicit a predominantly bottom-up stimulus-driven processing mode, characterised by increased gamma-band power in parietal, motor, frontal, and fronto-temporal cortices, as well as decreased beta-band power in regions dorsal to the Sylvian fissure. The complementary modulation of gamma- and beta-band activity aligns with prior findings on enhanced motor control, sensorimotor integration, and visuo-spatial processing during driving (Navarro et al., 2018; Sasai et al., 2016; Spiers & Maguire, 2007), thereby supporting H.2.

#### 3.1.3 Relationship between spatial oscillatory modulations

To investigate the antagonistic relationship between information processing modes and significant neural markers, we computed correlations between averaged cluster band power across conditions within participants.

During emotional speech, alpha- and beta-band cluster power were positively correlated ($r_{s}=.42$ , p<.001 ; df=39 ). Moreover, we observed negative correlations between gamma-band cluster power and alpha-band cluster power ($r_{s}=-.36$ , p<.001 ; df=39 ), as well as gamma- and beta-band cluster power ($r_{s}=-.28$ , p<.005 ; df=39 ).

In the workload conditions, gamma-band cluster power was significantly negatively correlated with beta-band cluster power ($r_{s}=-.51$ , p<.002 ; df=39 ). No other correlations reached significance for the main effect of visuo-spatial workload.

A control correlation analysis using the 3-minute resting-state MEG recording showed that the oppositely directed gamma- to alpha- and beta-band modulations were specific to the experimental manipulation. In contrast, during the resting state, we observed positive correlations between alpha-, beta-, and gamma-band power within the cluster vertices of the main effects (see Supplementary Material Section 10 for details).

To conclude, our correlation results suggest that information processing during socio-emotional speech and visuo-spatial workload is supported by antagonistic oscillatory activity, with alpha- and beta-band modulations linked to oppositely directed gamma-band modulations (Engel et al., 2001; Fries, 2005).

### 3.2 Co-modulation of valence and workload on information processing (R.2)

To address our second research question on the joint modulation of valence and workload on neural information processing (R.2; interaction effects), we applied MVPA using CSP combined with LDA to discriminate drives with combinations of low and high visuo-spatial workload and negative and positive socio-emotional speech in a four-class decoding task.

We assumed that the valence of speech and visuo-spatial workload co-modulate emotion appraisal and regulation. Under high workload, we expected that emotional appraisal would be inhibited and regulated (H.3). Motivated by findings from Kang et al. (2014) and Popov et al. (2012), which implicate prefrontal gamma as a key mechanism of regulatory control, we investigated whether modulations in frontal gamma-band activity are linked to the regulation of emotional interference and can distinguish the four conditions.

#### 3.2.1 Interaction effects revealing a third regulatory mode (H.3)

##### 3.2.1.1 Multivariate pattern analysis of the interaction between valence and visuo-spatial workload

Our results revealed clear discrimination among the four conditions in the test set, with performance well above the theoretical chance level (*F*1 score: 0.25), as indicated by an average *F*1 score of M=0.669 (95% CI : [0.619, 0.718] ), and a median of 0.655 (range: 0.30 to 0.95). Supplementary Figure S11 displays the test decoding performance for each participant. The averaged confusion matrix from the classifications revealed a cross-over interaction, with the most frequent misclassification occurring along the diagonal (see Supplementary Fig. S12B). This observation already hints at an underlying cross-over interaction between valence and visuo-spatial workload.

As a follow-up exploratory analysis, we examined whether inter-individual factors influence decoding performance using a median-split approach. The findings revealed that a larger proportion of female participants exhibited below-median decoding performance, which may point to gender-related differences during the integration of emotional and cognitive processes (Derntl et al., 2010; see Supplementary Material Section 11.1 for details).

We further explored whether decoding performance (low vs. high) affected the co-modulation of valence and workload on subjective measures and the IPA (H.3). While no effects emerged for subjective ratings, a significant three-way interaction for the IPA yielded evidence that only participants in the high decoding group displayed a valence-by-workload interaction, whereas the low decoding group did not. This pattern could reflect a connection between pupillary discontinuities (i.e., the IPA) and the ability to engage fronto-temporal mechanisms involved in emotional interference control (Supplementary Material Section 11.2 and Supplementary Figure S13).

##### 3.2.1.2 Localisation of informative neural dynamics from CSP components

**Fig. 5.**
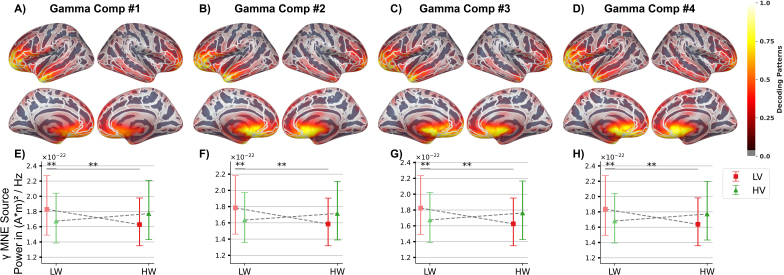
Decoding patterns of the four common spatial pattern components. *Note.* Components are used in the linear discriminant analysis (LDA). Panels (A) to (D) visualise the normalised values (range: 0 to 1) in the lateral (upper row) and medial view (lower row). Panels (E) to (H) display the source gamma-band power per condition for vertices exceeding the 90*^th^* percentile of the decoding pattern values. Significance levels from the Wilcoxon signed-rank test, corrected using false discovery rate: *** for p<.001 , ** for p<.01 , * for p<.05 . LV: low valence; HV: high valence; LW: low visuo-spatial workload; HW: high visuo-spatial workload.

To localise informative neural dynamics in the gamma band, the four CSP components were transformed into source space (Fig. 5A–D). This analysis identified the IFG, OFC, anterior temporal lobe, and mPFC as key regions underlying the joint modulation and interaction between emotional speech processing and attentional control during visuo-spatial workload. The decoding patterns were more pronounced in the left hemisphere, and the components exhibited highly similar spatial distributions in source space (Fig. 5A–D). Supplementary Figure S14 provides single-subject decoding patterns of the four components from four exemplary subjects, including two with high, one with average, and one with low decoding performance.

To examine the relationship between the decoding patterns and experimental conditions, we averaged source-level gamma-band power per condition across vertices exceeding the 90*^th^* percentile of the component values (Fig. 5E–H). Wilcoxon sign-rank tests revealed cross-over interactions, indicating a co-modulation between speech valence and visuo-spatial workload: During low visuo-spatial workload, source gamma-band power was significantly higher for drives with concurrent negative compared with positive speech (LV – HV LW – Component 1: $Z_{\left(40\right)}=-2.94$ , p=.005 ; Component 2: $Z_{\left(40\right)}=-3.01$ , p=.004 ; Component 3: $Z_{\left(40\right)}=-2.97$ , p=.005 ; Component 4: $Z_{\left(40\right)}=-3.03$ , p=.004 ). However, during high visuo-spatial workload, source gamma-band power significantly decreased for negative compared with positive speech (LV – HV HW – Component 1: $Z_{\left(40\right)}=-2.51$ , p=.015 ; Component 2: $Z_{\left(40\right)}=-2.40$ , p=.021 ; Component 3: $Z_{\left(40\right)}=-2.43$ , p=.019 ; Component 4: $Z_{\left(40\right)}=-2.46$ , p=.018 ).

While decoding increases sensitivity to effects affected by inter-individual neural variability, mass-univariate, permutation-based clustering is a commonly used complementary approach that identifies correlates in a data-driven manner at the group level (Maris & Oostenveld, 2007). To examine whether both approaches converge in their evidence, we performed a supplementary analysis with permutation-based clustering. This analysis revealed significant clusters of a cross-interaction in the gamma band (Supplementary Fig. S16). The identified gamma-band clusters comprised the OFC, ACC, and mPFC (lh: p=.03 ; rh: p=.012 ; Supplementary Fig. S16A). We performed post-hoc tests on gamma-band activity averaged across vertices within the significant cluster to compare the four conditions.

In line with our previous decoding results, the post-hoc tests revealed a significant cross-over interaction: During low workload, gamma power was significantly higher for negative speech than for positive speech (LV – HV LW: $Z_{\left(40\right)}=-3.18$ , p=.001 ). Across workload levels, gamma power decreased for negative speech (LV HW – LW: $Z_{\left(40\right)}=-3.82$ , p<.001 ) and increased for positive speech (HV HW – LW: $Z_{\left(40\right)}=-2.59$ , p=.009 ). Consequently, during high workload, gamma power was significantly lower for negative than for positive speech (LV – HV HW: $Z_{\left(40\right)}=-3.53$ , p<.001 ; Supplementary Fig. S16B).

Further supplementary analyses showed similar cross-interactions for the aperiodic broadband within the decoding patterns, and when not subtracting the aperiodic component from the gamma-band source power (Supplementary Figs. S15, S17, and S18).

##### 3.2.1.3 Subjective ratings

At the subjective level, we expected that interaction effects on regulatory control and information processing would also be reflected in a co-modulation of socio-emotional speech and visuo-spatial workload on perceived overall valence, arousal, effort, frustration, and distraction (H.3).

In line with this hypothesis, linear mixed-effects models revealed that perceived valence, arousal, and frustration were jointly modulated by the combination of emotional speech and visuo-spatial workload (interaction effect; valence: $F_{\left(2,206.49\right)}=4.40$ , p=.013 ; excluded outlier observations: 11.63% ; arousal: $F_{\left(2,222.00\right)}=3.57$ , p=.030 ; excluded outlier observations: 5.11% ; frustration: $F_{\left(2,220.85\right)}=7.150$ , p<.001 ; excluded outlier observations: 5.49% ). Contrary to our hypothesis, we did not observe a significant interaction effect on perceived effort and distraction.

**Fig. 6.**
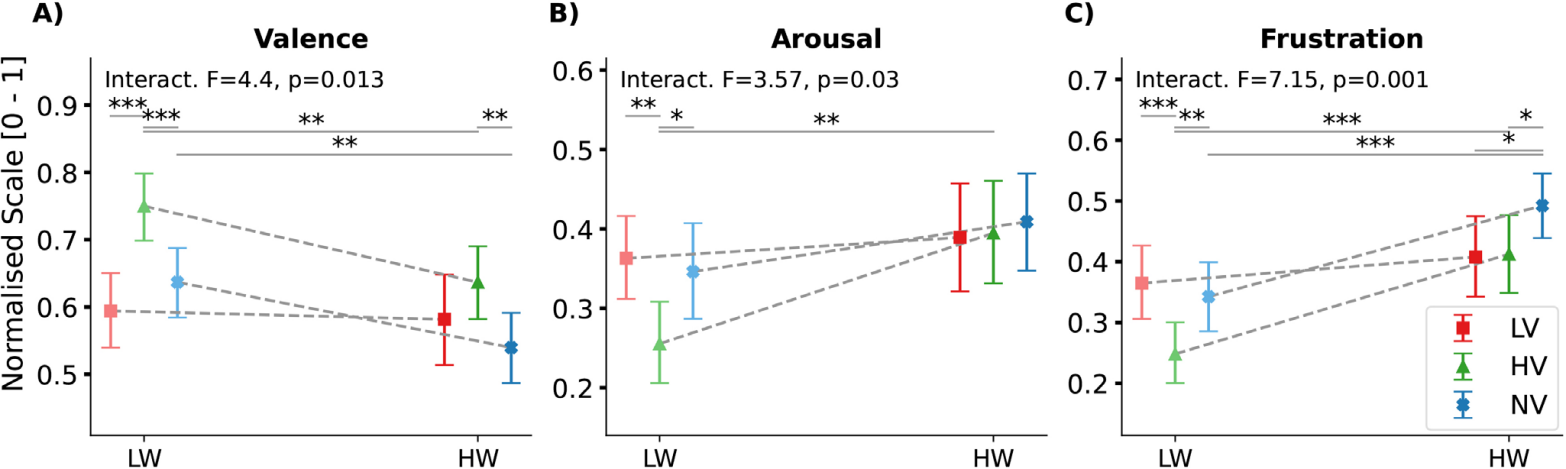
Post-hoc comparisons of interaction effects between emotional speech and visuo-spatial workload in ratings of perceived (A) valence, (B) arousal, and (C) frustration. *Note.* Coloured symbols and error bars represent the bootstrapped grand averages and their Bonferroni-corrected 2.5*^th^* and 97.5*^th^* confidence interval (CI) across participants. LV: low valence; NV: neutral valence; HV: high valence; LW: low visuo-spatial workload; HW: high visuo-spatial workload. Significance level from the Wilcoxon signed-rank test corrected with false discovery rate: *** for p<.001 , ** for p<.01 , * for p<.05 .

Next, we investigated the nature of the significant interactions using post-hoc tests. These showed that valence ratings were significantly lower in the negative than in the positive speech condition under low workload (LV – HV LW: $Z_{\left(47\right)}=-4.39$ , p<.001 ; Fig. 6A). The difference was absent under high visuo-spatial workload (n.s.), due to a significant decrease in perceived valence for drives with positive speech (HV HW – LW: $Z_{\left(47\right)}=-3.55$ , p=.001 ). For drives with neutral speech, workload significantly reduced perceived valence (NV HW – LW: $Z_{\left(47\right)}=-3.39$ , p=.002 ). Arousal and frustration ratings were significantly higher for drives with negative than with positive speech under low workload (arousal LV – HV LW: $Z_{\left(47\right)}=-3.23$ , p=.006 ; frustration LV – HV LW: $Z_{\left(47\right)}=-3.83$ , p<.001 ; Fig. 6B, C). Furthermore, low workload drives with positive speech were significantly lower rated in arousal than those with neutral speech (HV – NV LW: $Z_{\left(47\right)}=-2.58$ , p=.029 ). From low to high workload, perceived arousal ($Z_{\left(47\right)}=-3.77$ , p=.002 ) and frustration ($Z_{\left(47\right)}=-4.00$ , p<.001 ) increased for drives with positive speech, eliminating any differences between the positive and negative speech conditions under high workload. Finally, perceived frustration during neutral speech increased with workload (NV HW – LW: $Z_{\left(47\right)}=-4.26$ , p<.001 ), leading to significantly higher frustration for high workload drives with neutral compared with negative speech (LV – NV HW: $Z_{\left(47\right)}=-2.47$ , p=.021 ).

#### 3.2.2 Interim summary of the regulatory mode

In conclusion, the subjective findings and MEG MVPA revealed a joint modulation of socio-emotional speech valence and visuo-spatial workload on subjective emotional appraisal and neural regulatory mechanisms.

As hypothesised, participants attended and subjectively appraised positive speech but only during low visuo-spatial workload (H.3; Fig. 6). Increased workload diminished the perceived positivity of positive speech, which rendered responses to positive speech more similar to those elicited by negative speech. Interestingly, the experienced effort was affected by the driving-related workload and emotional speech separately, but not by their combination. At the neural level, oscillatory gamma activity in the OFC, temporal pole, and mPFC increased for positive speech under high workload, indicating greater engagement of inhibitory control mechanisms (H.3; Fig. 5).

Subjective ratings showed that experiences of negative speech were unaffected by workload, implying that negative speech was down-regulated regardless of workload level (Fig. 6). However, the decrease in fronto-temporal gamma activity indicates that top-down regulation of negative speech weakened under high visuo-spatial workload (Fig. 5).

## 4 Discussion

The MEG–eye-tracking study addressed two research questions: (1) What are the distinct neural signatures of socio-emotional speech processing and visuo-spatial cognition during the dual task of driving and listening (R.1) and (2) do the valence of socio-emotional speech and level of visuo-spatial workload jointly modulate information processing (R.2)?

To investigate these questions in a near-realistic scenario, we employed MEG, eye tracking, a driving simulator, and naturalistic emotional speech stimuli from the validated GAUDIE database (Lingelbach et al., 2024). Using a dual-task paradigm involving driving under varying visuo-spatial workload and concurrent emotional speech listening, we hypothesised three distinct processing mechanisms. Socio-emotional speech processing was expected to decrease gamma-band power in parietal regions and increase beta-band power associated with predictive listening (R.1). In contrast, increased visuo-spatial workload was hypothesised to result in decreased beta-band and increased gamma-band power, along with increased pupil dilation (R.1). A third, regulatory processing mode was postulated to reflect emotion regulation through frontal gamma-band activity. Specifically, we hypothesised that positive emotional speech would be attended to and appraised under low, but not high, visuo-spatial workload. Under high workload, gamma-band-based regulatory activity was expected to suppress emotional interference to support goal-directed processing associated with the driving task (R.2).

Our findings revealed distinct neural, gaze-related, and behavioural signatures distinguishing antagonistic information processing modes for socio-emotional speech processing and visuo-spatial cognition (R.1). In addition, subjective ratings indicated that speech valence and visuo-spatial workload co-modulated both inhibitory control and emotional appraisal of positive speech (R.2). Specifically, emotional appraisal of positive speech occurred only under low, but not high, visuo-spatial workload. At the neural level, the multivariate pattern analysis revealed a co-modulatory effect of speech valence and visuo-spatial workload on fronto-temporal gamma-band activity (R.2). Gamma oscillations in the OFC, temporal pole, and mPFC indicated reduced inhibitory control for positive compared with negative speech under low workload, and increased inhibitory control for positive compared with negative speech under high workload. Negative speech triggered the regulatory mode even during low workload; however, the effectiveness of top-down control appeared to diminish as workload increased.

The study offers converging evidence for distinct processing modes underlying socio-emotional speech processing, visuo-spatial cognition, and regulatory control mechanisms that inhibit emotional interference as a function of available cognitive resources in complex, naturalistic environments. The findings also revealed circumstances under which socio-emotional speech valence and visuo-spatial workload jointly influence cognitive resource allocation and regulatory mechanisms.

### 4.1 Oscillatory dynamics guiding information processing in multisensory environments

#### 4.1.1 Emotional speech processing is linked to predictive listening and socio-emotional cognition

Consistent with our hypothesis on the role of beta- and gamma-band oscillations during socio-emotional speech listening, we found that increased parietal beta- and decreased gamma-band power served as distinct markers of socio-emotional compared with neutral speech processing. Additionally, we observed modulations in alpha-band power in temporo-parietal regions.

The spatial distribution of the beta-band cluster (Fig. 2B) is in concordance with the previously described dorsal stream of deliberate emotion processing (Phillips et al., 2003; Tucker et al., 1995). We did not observe significant clusters in ventral stream areas associated with sensory processing and automatic emotion processing (Phillips et al., 2003; Schirmer & Kotz, 2006; Tucker et al., 1995).

One possible explanation is that the dual task, characterised by distributed cognitive resources, may have interfered with low-level acoustic analysis involved in automatic emotion recognition (ventral processing stream; Schirmer & Kotz, 2006), instead engaging dorsal regions associated with deliberate emotion processing and later evaluative stages (Phillips et al., 2003; Tucker et al., 1995). Previous linguistic research has associated increased parietal beta activity with top-down guided modulation of information flow and the updating of content-related predictions (Arnal & Giraud, 2012; Sedley et al., 2016), as well as with the reactivation of content representations during speech perception (Spitzer & Haegens, 2017; Zioga et al., 2023). Such top-down influences are proposed to be particularly important during socio-emotional interactions (Grandjean, 2021).

Particularly in line with the latter account, we observed increased oscillatory beta-band power and decreased gamma-band power in temporo-parietal regions, including the precuneus, cuneus, and TPJ, in the main effect of emotional speech (Fig. 2B). This functional network has been linked to mentalising and cognitive ToM (Saxe & Kanwisher, 2003; Schurz et al., 2021) and is likely coordinated by beta oscillations to facilitate predictive listening as well as inferential and evaluative processes in socio-emotional contexts (see also Mossad et al., 2022). In contrast, purely informative, neutral speech, such as weather forecasts, appears to be less conducive to these top-down guided modulations.

In their electroencephalography study, Pinheiro et al. (2017) reported enhanced predictive processing reflected in increased beta-band activity exclusively for positive but not negative vocalisations. In contrast, our findings reveal such enhancement for both positive and negative speech sequences. Importantly, Pinheiro et al. (2017) employed brief, context-free vocalisations, such as laughter and growls. Such stimuli may have complicated predictions and elicited weaker effects than coherent, naturalistic speech sequences (Hagoort, 2019; Hamilton & Huth, 2020).

In addition to the beta-band clusters, we observed clusters in the alpha and gamma bands for the main effect of emotional speech. These encompassed parietal regions, including the superior parietal lobe, precuneus, and cuneus, as well as the posterior cingulate cortex and posterior STS. They have previously been identified as the network relevant to driving, particularly for spatial attention and processing (Haghani et al., 2021; Palmiero et al., 2019). In agreement with previous studies studying speech processing during driving (Fort et al., 2010; Sasai et al., 2016; Sonnleitner et al., 2012), we propose that reduced gamma-band power, together with increased alpha-band power in this driving network, reflects diminished spatial cognition. This implies that cognitive resources are reallocated from driving-related processing towards emotional speech processing.

The subjective ratings and correlation analyses also support this interpretation. Emotional speech stimuli increased distractions. Likewise, higher speech arousal, also characterising the emotional speech stimuli, was correlated with greater effort and distraction during the driving task.

To summarise, listening to emotional speech likely triggered both enhanced anticipatory listening and socio-emotional cognition. At the same time, visuo-spatial attention and processing decreased. We propose that these are signatures of a top-down guided processing mode during socio-emotional speech listening (resembling an internalised processing; Oosterwijk et al., 2015).

#### 4.1.2 Visuo-spatial cognition and sensorimotor processing increase during difficult drives

Our results indicate that increased visuo-spatial workload elicits enhanced bottom-up stimulus-driven processing. Under high visuo-spatial workload and increased task demand, we observed decreased driving quality as well as increased subjective effort, pupil dilation, and visual tunnelling, reflected in reduced gaze dispersion (Nunes & Recarte, 2002, confirming H.2).

We proposed that modulations in pupil diameter index attentional effort during visuo-spatial attention and the tracking of multiple objects. Translational animal, stimulation, and neuroimaging studies have demonstrated that pupil dilation is linked to neural circuits involved in attention and visuo-spatial processing (Alnæs et al., 2014; Joshi et al., 2016; Vinck et al., 2015). These circuits include the nucleus locus coeruleus and associated noradrenergic system (Alnæs et al., 2014; Joshi et al., 2016; Vinck et al., 2015), the oculomotor system including the frontal eye fields and pathways to the superior colliculus (Alnæs et al., 2014; Joshi et al., 2016), cingulate cortex (Joshi et al., 2016), and superior parietal lobule (Alnæs et al., 2014).

At the neural level, we observed increased gamma-band power in parietal, motor, frontal, and fronto-temporal cortices during high compared with low visuo-spatial workload, while beta-band power decreased in regions dorsal to the Sylvian fissure (H.2). The spatial distribution of the gamma- and beta-band clusters partially overlaps with neural circuits linked to pupil dilation and aligns with findings from previous driving studies. These studies reported increased activation in motor and parietal regions during challenging driving scenarios, suggesting that these areas contribute to motor control, sensorimotor integration, and visuo-spatial processing (Navarro et al., 2018; Sasai et al., 2016; Spiers & Maguire, 2007). The antagonistic relationship between gamma- and beta-band power in the motor cortex and related areas, such as the frontal eye fields, has been well documented in electrocorticography (ECoG; e.g., Crone et al., 1998), stimulation (e.g., Joundi et al., 2012), and animal studies (e.g., Sendhilnathan et al., 2017).

Shi et al. (2023) offered a further interpretation of the role of gamma-band oscillations during challenging driving scenarios. The authors outlined in their review that OFC gamma-band activity is associated with the self-monitoring of decision making in social contexts (Shi et al., 2023). Since the high visuo-spatial workload condition included unpredictable car agents, increased prefrontal gamma activity likely reflects driving-related decision making (e.g., lane changes), as well as social evaluation and predictive processes in response to the other agents. Beta-band power, which was negatively correlated with gamma-band power, decreased in temporal and parietal regions, including the STG, right TPJ, and right supramarginal gyrus. This finding is in line with an MEG study of van Pelt et al. (2016). Using Granger causality, van Pelt et al. (2016) investigated the relationship between antagonistically operating beta- and gamma-band oscillatory activity in the context of predictive coding during the observation of animated bowling actions. In their study, oscillatory power in the TPJ and mPFC increased in the gamma band and decreased in the beta band during prediction errors. The authors proposed that predictive coding involves bottom-up sensory encoding, driven by gamma-band modulations, and top-down prediction evaluation, driven by beta-band modulations (van Pelt et al., 2016).

In summary, we observed oscillatory signatures indicative of bottom-up stimulus-driven processing and the reallocation of cognitive resources to the driving task under high visuo-spatial workload. The gamma–beta oscillatory pattern suggests compensatory processes involving predictive cognition and motor responses. These compensatory processes are likely recruited to meet increased task demands and associated cognitive strain, as also indicated by greater pupil dilation.

#### 4.1.3 Emotional regulation through fronto-temporal gamma oscillatory activity is co-modulated by valence and workload

Our second research question and last hypothesis (H.3) target the co-modulation of socio-emotional speech valence and workload (i.e., interaction effects). We hypothesised that appraisal and processing of positive emotion and, thus, valence-specific signatures, would occur only when sufficient cognitive resources are available under low visuo-spatial workload (Baddeley, 1992; Cowan, 2017; Lingelbach & Rieger, 2025; Wickens, 2014). Under high visuo-spatial workload, prefrontal gamma oscillatory activity was expected to regulate emotional interference (Kang et al., 2014; Popov et al., 2012; Quirk & Beer, 2006).

Our findings largely supported the hypothesis and offered even further insights. A neural signature in the form of prefrontal and fronto-temporal gamma-band activity was co-modulated by the valence of speech and workload (cross-over interactions). In line with previous studies (Kang et al., 2014; Popov et al., 2012), we propose that this fronto-temporal signature is related to the regulatory processing of emotional interference. Under low workload, fronto-temporal gamma-band activity was greater for negative than for positive speech (in line with H.3). Under high visuo-spatial workload, fronto-temporal oscillatory gamma activity increased for positive speech, and decreased for negative speech (reversed pattern).

The gamma-band signature included the OFC, temporal pole, mPFC, and ACC. These regions were previously linked to top-down guided cognitive control (Koechlin et al., 2003) and emotion regulation (see Quirk & Beer, 2006, for review; see Berboth & Morawetz, 2021; and Morawetz & Basten, 2024, for meta-analyses). The spatial distribution of the gamma-band signature partially overlaps with that observed under increased visuo-spatial workload, suggesting that these regions also contribute more generally to the coordination of goal-directed processes (Koechlin et al., 2003).

Emotion regulation describes the capacity to control and modify emotional processing through implicit or explicit strategies, such as distraction, reappraisal, and suppression (J. J. Gross, 1998). Using intracranial electroencephalography (iEEG) and an emotional picture viewing task, Sonkusare et al. (2023) proposed gamma activity in the mPFC and OFC to regulate negative emotions (see also Golkar et al., 2012; Popov et al., 2012; Quirk & Beer, 2006). Functional MRI studies indicate that emotion regulation by the mPFC, vlPFC, OFC, and temporal pole is likely achieved through inhibition of limbic emotion-related processing in the insula and amygdala (Berboth & Morawetz, 2021; Goldin et al., 2008; Golkar et al., 2012; Ochsner et al., 2012; Sonkusare et al., 2020). Lesion studies also reported that damage to the OFC impairs the ability to modulate or inhibit neural responses to aversive stimuli (Hooker & Knight, 2006; Rule et al., 2002). Moreover, the OFC and mPFC are involved in decision making, self-reflection, and cognition in social contexts (Amodio & Frith, 2006; Bechara et al., 2000; see Van Overwalle, 2009, for meta-analysis).

In light of these previous findings (e.g., Kang et al., 2014; Morawetz & Basten, 2024; Popov et al., 2012; Van Overwalle, 2009), our results suggest that increased fronto-temporal gamma-band activity contributes to the inhibition of emotional responses to negative speech under low workload (see Lingelbach et al., 2023, for similar findings when using continuous naturalistic speech; but cf. Mothes-Lasch et al., 2012, using an event-related study with negative words). However, positive speech was emotionally perceived and appraised under low workload. The subjective ratings in our study support this interpretation, revealing higher valence, as well as lower arousal and frustration for positive relative to negative speech, but only under low visuo-spatial workload.

Under high workload, gamma-band activity increased in response to positive speech. In line with H.3, we interpret this as active emotion regulation of positive speech due to now limited available processing resources. However, contrary to our expectations, fronto-temporal gamma activity decreased for negative speech at high workload. This finding has two potential implications: First, regulating negative speech appears to demand greater cognitive resources (Cromheeke & Mueller, 2014; Lingelbach & Rieger, 2025; Schweizer et al., 2019). Second, at high workload, these resources seem to become depleted, thereby compromising inhibitory control (Cowan, 2017; Wickens, 2014). Despite this, subjective ratings and driving behaviour in response to negative speech remained unaffected by workload.

##### 4.1.3.1 Gaze signatures sensitive to the emotion–cognition interaction

In line with Vogels et al. (2018), we observed clear distinctions between modulations in pupil diameter and discontinuities in pupil diameter changes. This suggests that these gaze-related measures may index different cognitive processes. While pupil dilation increased under high visuo-spatial workload (confirming H.2), discontinuities in pupil diameter changes remained unaffected. Contrary to our hypothesis that discontinuities in changes would be jointly modulated by speech valence and workload level, we found no significant co-modulation. However, exploratory analysis indicated that discontinuities in pupil diameter changes may be partially linked to the fronto-temporal gamma-band signature involved in regulating emotional interference. This preliminary observation warrants further investigation. To summarise, our findings support the notion that the pupillary measures index distinct cognitive processes. While pupil dilation appears to reflect attentional effort and the required cognitive resources to perform a task (Van der Wel & Van Steenbergen, 2018), the frequency of pupillary discontinuities may relate to attentional control involved in allocating or redistributing cognitive resources (Demberg & Sayeed, 2016; Vogels et al., 2018).

##### 4.1.3.2 Complementary approach of multivariate pattern and mass-univariate analysis

Mass-univariate statistics such as permutation-based clustering examine activation from clusters separately (Haufe et al., 2014), and assume that neural activation patterns are similarly localised across subjects (Holdgraf et al., 2017; Marsicano et al., 2024). Consequently, they may be substantially limited when investigating individual-specific processing strategies (Marsicano et al., 2024) or naturalistic stimulus material (Sonkusare et al., 2019). MVPA enhances sensitivity by integrating measurement sources within a multidimensional framework, while accounting for inter-individual neural variability (Haufe et al., 2014; Holdgraf et al., 2017; Marsicano et al., 2024). Our complementary approach, incorporating MVPA, provided novel insights into the role of fronto-temporal gamma-band oscillatory activity in the integration and interaction of socio-emotional speech processing and visuo-spatial cognition.

In summary, our findings suggest that participants attended and appraised positive speech but only during low visuo-spatial workload. They aimed to inhibit negative speech regardless of the current workload level. However, the effectiveness of the top-down guided emotional regulation of negative speech declined as workload increased. This observation has important practical implications: As workload increases further, listening to negative speech may have a greater detrimental impact on driving performance than positive speech.

### 4.2 Studying the brain in its natural environment

We employed a multimodal approach, combining eye tracking and MEG, alongside a near-realistic experimental paradigm, to investigate effects of and interactions between cognitive and emotional processing in a driving simulator study. The experimental dual-task paradigm incorporated both a driving simulation, which mirrored authentic driving experiences, and naturalistic speech. In contrast to the only other available German emotional speech database (which includes 10 short sentences spoken with varying emotional prosody by professional actors; Burkhardt et al., 2005), the GAUDIE database features longer conversational speech with coherent context. Furthermore, it was developed and validated to explicitly induce positive, neutral, and negative emotions. Consequently, its emphasis lies on *how* participants emotionally experience the stimulus (Lingelbach et al., 2024), rather than solely on *what* valence the stimulus possesses (Burkhardt et al., 2005).

Naturalistic, ecologically valid research is expected to enhance transferability to real-world contexts, as it involves a lower degree of abstraction than studies using non-natural, isolated stimuli (Hamilton & Huth, 2020). Previous comparisons of linguistic studies have revealed processing differences between natural and isolated speech in terms of lateralisation and specialisation. Furthermore, our results suggest that continuous negative speech is already inhibited during low workload (see also Lingelbach et al., 2023), whereas studies using single words presented in an event-related design did not report emotion inhibition for negative words during low load (Mothes-Lasch et al., 2012).

According to the criteria defined by Hamilton and Huth (2020), our study demonstrates a high level of ecological validity. A German online survey of 1,700 participants indicated that 67% regularly listen to audio content while travelling, covering 82% of their total travel time when driving (Landesanstalt für Medien NRW, 2021). This underscores both the representativeness of the chosen paradigm and the practical relevance of understanding brain function and cognition during the dual task of driving while listening to speech or engaging in emotionally loaded in-car conversations. In real-life driving situations, additional sources of emotional responses, such as dangerous or rule-breaking manoeuvres performed by other drivers, can further impact cognitive processes. Therefore, future research should examine how different types of emotionally charged events affect the integration of emotional and cognitive processes, as well as the regulation of emotional responses during driving.

### 4.3 Limitations

Several shortcomings of this study should be noted.

Although we chose a fully equipped driving simulator with authentic driving scenarios, the experience of driving in the MEG remains considerably different from real-world driving. To minimise muscle artefacts, participants’ upper body and arm movements were restricted with pillows during steering, and braking was performed with the left foot. Future studies employing EEG should aim to replicate the cortical signatures observed here in real-world, out-of-laboratory driving settings. Such EEG studies should further investigate whether midfrontal theta-band oscillatory activity, related to executive functioning during driving (Wascher et al., 2018), is co-modulated by socio-emotional speech and visuo-spatial workload. While midfrontal theta is reliably detected with EEG, these signals primarily originate from superficial radial dipole layers, to which MEG is less sensitive (Srinivasan et al., 2006).

We observed a co-modulation of socio-emotional speech valence and workload on fronto-temporal gamma oscillations, reflected in a significant cross-over interaction in the clustering analysis when localising oscillatory source power using MNE (R.2), but not when using DICS (R.1). One possible explanation for the discrepancy between the two localisation methods is that DICS may have partially filtered out oscillatory changes related to emotion regulation (J. Gross et al., 2001; van Vliet et al., 2018), if participants had already engaged inhibitory control during baseline periods to detach from preceding emotional experiences.

Although naturalistic speech offers a more holistic emotional experience, disentangling the individual contributions of semantic content, paralinguistic features, and their interaction remains challenging in our study (but cf. Alday, 2019, for study approaches with natural stimuli). Hence, they likely contributed to the observed neurophysiological signatures during the dual task. Future research could examine oscillatory modulations and magnetic fields time locked to salient events to further elucidate the interaction between emotional and cognitive processes in naturalistic experimental paradigms (i.e., salient acoustic events, Khalighinejad et al., 2017; but also events linked to eye blinks, Alyan et al., 2023; and object pursuits, Agtzidis et al., 2020).

## 5 Conclusion

In the study, we identified antagonistic neural processing modes for socio-emotional speech and visuo-spatial cognition during the dual task of speech listening and driving, based on permutation-based clustering of oscillatory source power. Increased beta- and decreased gamma-band source power in parietal regions indicated an internalised processing mode for socio-emotional speech. This mode is likely associated with enhanced anticipatory listening, social cognition, and reduced spatial processing. In contrast, decreased beta-band and increased gamma-band source power, along with increased pupil dilation, signalled an externalised bottom-up processing mode under high visuo-spatial workload. This likely reflects enhanced sensorimotor processing and spatial cognition, but also increased cognitive strain due to heightened task demands.

Multivariate pattern analysis and subjective ratings indicated emotional appraisal of positive speech only under low, but not high, visuo-spatial workload. Under high visuo-spatial workload and reduced cognitive resources, gamma-band activity in the OFC, temporal pole, and mPFC appears to mediate the regulation of emotional speech. Participants attempted to inhibit negative speech regardless of workload level; however, the effectiveness of top-down emotional regulation of negative speech declined as workload increased.

To conclude, by combining neural, gaze, and behavioural correlates, we found converging evidence of distinct information processing modes for visuo-spatial cognition and auditory socio-emotional speech processing in naturalistic multisensory environments. Lastly, our multivariate analyses revealed a gamma-based top-down regulatory signature in fronto-temporal regions. This mechanism flexibly up- and down-regulates emotional speech processing in response to potential audio-visual interference in complex, naturalistic environments.

## Data and Code Availability

Data and code supporting the findings of this manuscript are available upon request via the OSF project (https://osf.io/um6vw/).

## Supplementary Material

Supplementary Material
