# Supplementary material for: Oscillatory and gaze signatures of socio-emotional speech processing, visuo-spatial cognition, and their interaction in a near-realistic dual-task MEG study
Source: Imaging Neurosci (Camb). 2026 Feb 17;4:IMAG.a.1134. doi: 10.1162/IMAG.a.1134 (PMC12915007; doi:10.1162/IMAG.a.1134)
Supplement: Supplementary Material [file IMAG.a.1134_supp.pdf]

Supplementary Material: Oscillatory and Gaze Signatures of Socio-Emotional Speech Processing, Visuo-Spatial Cognition, and Their Interaction in a Near-Realistic Dual-Task MEG Study

Katharina Lingelbach[1,2*], Christoph S. Herrmann[3,4,5] and Jochem W. Rieger[1]

[1] Applied Neurocognitive Psychology, Department of Psychology, Carl von Ossietzky Universität Oldenburg,

Küpkersweg 74, 26129 Oldenburg, Germany

[2]Applied Neurocognitive Systems, Fraunhofer Institute for Industrial Engineering IAO,

Nobelstraße 12, 70569 Stuttgart, Germany

[3] Experimental Psychology Lab, Department of Psychology, Carl von Ossietzky Universität Oldenburg,

Ammerländer Heerstr. 114-118, 26129, Oldenburg, Germany

[4] Cluster for Excellence "Hearing for All", Carl von Ossietzky Universität Oldenburg,

Carl-von-Ossietzky-Straße 9-11, 26129, Oldenburg, Germany

[5] Research Center Neurosensory Science, Carl von Ossietzky Universität Oldenburg,

Ammerländer Heerstr. 114-118, 26129, Oldenburg, Germany

*Correspondence: katharina.lingelbach@uni-oldenburg.de

January 9, 2026

1 Stimulus Set From the German AUDItory Emotional Database

We excluded four audio sequences of the neutral condition (i.e., items 3, 5, 8, and 10) from the database to equalise the number of stimuli per condition. Those stimuli were chosen due to their larger confidence interval in their valence ratings in the validation study. The stimuli were previously rated in valence and arousal using a scale between 0 to 100 (Lingelbach et al., 2024). The final negative stimulus set in this study exhibited an average valence of 21.44 ($SD = 10.72$, $min = 2.10$, $max = 53.06$), and an average arousal of 74.21 ($SD = 13.89$, $min = 25.03$, $max = 100.00$). The average valence of neutral stimuli was 50.17 ($SD = 11.29$, $min = 5.46$, $max = 82.53$), while the average arousal was 29.18 ($SD = 18.79$, $min = 0.00$, $max = 77.17$). For positive stimuli, the average valence was 71.15 ($SD = 17.29$, $min = 9.76$, $max = 100.00$), and the average arousal was 57.87 ($SD = 20.95$, $min = 3.17$, $max = 100.00$).

In this study, the valence and arousal ratings of the speech stimuli significantly differed between emotional speech conditions (speech valence: $F_{(2,\ 224.43)} = 89.72$, $p < .001$; excluded outlier observations: $5.49\ \%$; speech arousal: $F_{(2,\ 222.37)} = 22.87$, $p < .001$; excluded outlier observations: $5.49\ \%$; see also Supplementary Figure 3C – D), but not between the workload levels. Participants rated positive stimuli significantly higher in valence than neutral (HV – NV: $M = 0.29$, $95\ \%\ CI\ [0.23,\ 0.35]$; $Z_{(47)} = -5.67$, $p < .001$), and negative stimuli (LV – HV: $M = -0.30$, $95\ \%\ CI\ [-0.37,\ -0.22]$; $Z_{(47)} = -4.99$, $p < .001$). Arousal ratings were significantly higher for negative stimuli compared to neutral (LV – NV: $M = 0.16$, $95\ \%\ CI\ [0.09,\ 0.23]$; $Z_{(47)} = -3.71$, $p < .001$), and positive stimuli (LV – HV: $M = 0.17$, $95\ \%\ CI\ [0.11,\ 0.24]$; $Z_{(47)} = -4.53$, $p < .001$). No significant main effects of visuo-spatial workload or further significant pairwise contrasts of emotional speech (valence LV – NV and arousal HV – NV) were observed.

2 Analysis of Conversation Content Recall and Misses

Linear mixed-effects models revealed a significant main effect of emotional speech on recall ($F_{(2,\ 225.95)} = 37.18$, $p < .001$; excluded outlier observations: $4.73\ \%$) and content misses ($F_{(2,\ 223.49)} = 12.23$, $p < .001$; excluded outlier observations: $3.60\ \%$), in responses to the conversation content questions answered at the end of each driving block (Supplementary Figure 3E – F). Content misses were significantly more frequent for neutral compared to negative conversations (LV – NV: $M = -0.09$, $95\ \%\ CI\ [-0.14,\ -0.04]$; $Z_{(47)} = -3.15$, $p = .005$) and positive conversations (HV – NV: $M = -0.11$, $95\ \%\ CI\ [-0.18,\ -0.04]$; $Z_{(47)} = -2.75$, $p = .008$). Conversely,

recall performance was significantly lower for negative compared to neutral conversations (LV – NV: $M = -0.19$, 95 % CI $[-0.23, -0.14]$; $Z_{(47)} = -5.25$, $p < .001$), and for negative compared to positive conversations (LV – HV: $M = -0.23$, 95 % CI $[-0.27, -0.18]$; $Z_{(47)} = -5.53$, $p < .001$).

3 Analysis of Movement-Related Independent Components Removal

To evaluate how well the manually identified contaminated independent components (ICs) corresponded to muscle- and movement-related artefacts in the MEG sensor data, we conducted two follow-up analyses. First, we compared the maximum absolute cross-correlation between behavioural driving signals (i.e., the change rates of the steering wheel, brake, and acceleration pedal) and the time series of the excluded ICs with the corresponding correlations obtained for the retained ICs. Second, we evaluated the impact of removing muscle-related ICs on the correlation strength between the behavioural driving signals and MEG magnetometer channels positioned over motor regions corresponding to the hands and the left and right foot.

In both analyses, we computed the maximum absolute cross-correlation between the behavioural driving signals and independent component time courses (Analysis I) as well as MEG magnetometer channels (Analysis II) within a $\pm$ 200 ms lag window. First, the first temporal derivative was computed to capture rapid movement-related fluctuations, and both signals were subsequently mean-centred. Afterwards, the two time series were aligned and samples with missing values removed. Finally, we computed the maximum absolute cross-correlation within the specified lag range and normalised it by the product of the standard deviations of the two signals and the number of samples, yielding a coefficient confined to the interval -1 and 1.

In the first analysis, we averaged the correlation coefficients across the driving metrics (steering wheel, acceleration pedal, and brake pedal) separately for the excluded and included components, and statistically evaluated the difference in correlation strength using a Wilcoxon signed-rank test (scipy version 1.5.0). We observed a significant difference, with higher correlation coefficients for the excluded ICs compared to the retained ICs, $Z_{(47)} = -3.12$, $p = .001$. The bootstrapped mean of the contrast excluded – retained ICs was $r = .001$, 95 % CI $[.000, .001]$. The bootstrapped mean of the maximum absolute cross-correlation across subjects between the driving signals and the IC most strongly reflecting the movement-related artefact was $r = 0.013$, 95 % CI $[0.012, 0.015]$ for the first derivative of the acceleration signal, $r = 0.011$, 95 % CI $[0.010, 0.014]$ for the brake signal, and $r = 0.015$,

$95\ \%\ CI\ [0.013,\ 0.017]$ for steering-wheel signal.

In the second analysis, we selected magnetometer channels over the left hemisphere corresponding to the right-foot area for the acceleration signal (MEG0641, MEG0621); channels over the right hemisphere corresponding to the left-foot area for the brake signal (MEG1031, MEG0621); and bilateral hand-region channels for the steering wheel signal (MEG1241, MEG1231, MEG0331, MEG0321). For each driving metric, correlation coefficients from the raw (before IC removal) and cleaned (after IC removal) MEG data were averaged across the respective magnetometer channels, and differences in correlation strength were assessed using Wilcoxon signed-rank tests. The results showed a significant reduction in correlation strength following IC removal across all three driving metrics (acceleration and right-foot area: $Z_{(47)} = -3.14, p = .001$; After – Before: $r = -.001$, $95\ \%\ CI\ [-.001,\ -.000]$; brake and left-foot area: $Z_{(47)} = -2.55, p = .010$; After – Before: $r = -.001$, $95\ \%\ CI\ [-.001,\ -.000]$; steering wheel and hand areas: $Z_{(47)} = -3.27, p = .001$; After – Before: $r = -.001$, $95\ \%\ CI\ [-.001,\ -.000]$).

Together, the findings indicate that the identified ICs reflected movement- and muscle-related artefacts, and that their removal significantly reduced artefactual contributions to the MEG signals. Supplementary Figure 1 provides an illustrative example of the acceleration-pedal time series, the raw and cleaned MEG signal from magnetometer channel MEG0641 positioned over the right-foot representation of the left motor cortex, and the excluded IC most strongly corresponding to muscle-related artefacts.

4 Centre Frequencies of the Frequency Bands

Supplementary Table 1

Descriptive Statistics of Centre Frequencies per Power Band in Hz

Frequency Band	Mean	Standard Deviation	Minimum	Maximum
Alpha	10.29	0.51	9.22	11.46
Beta	19.80	0.25	19.23	20.52
Gamma	33.59	0.78	32.29	35.25

Note. Centre frequencies were derived from the resting state sensor power through power parametrisation using the FOOOF algorithm (Donoghue et al., 2020).

5 Common Spatial Patterns for Multiclass Decoding

Common spatial pattern (CSP) analysis provides a data-driven, supervised signal decomposition that extracts components maximally discriminative in signal variance between experimental conditions. It computes spatial filters that maximise variance in one class (i.e., experimental condition), while reducing it in others (Blankertz et al., 2008). Its extension to multiclass decoding is achieved via joint approximate diagonalisation (JAD) of class-wise covariance matrices (Grosse-Wentrup & Buss, 2008). JAD estimates a transformation matrix that approximately diagonalises these covariance matrices across all classes. The columns of the obtained transformation matrix serve as spatial filters and are ranked to maximise the mutual information between the extracted components and class labels. Each column of the transformation matrix defines a spatial filter. Applying these filters to the data yields components, which are subsequently ranked by their mutual information with the class labels (Grosse-Wentrup & Buss, 2008). These CSP components serve as features for subsequent machine learning classification, for example, using Linear Discriminant Analysis (LDA), to distinguish between the different classes. Similar to the CSP, LDA projects the input data onto a linear subspace that maximises separation between classes.

6 Calculation of Gaze Dispersion, Pupil Diameter in Millimetres, and Pupillary Activity Index

6.1 Gaze Dispersion

To quantify gaze dispersion, we first computed the gaze centroid (x_c, y_c) as the x- and y-coordinates averaged across all fixations (x_i, y_i: horizontal and vertical coordinates of fixation i) within the block. Fixations were weighted by their duration (d_i: duration of fixation i).

$$x_c = \frac{\sum_i d_i x_i}{\sum_i d_i}, \quad y_c = \frac{\sum_i d_i y_i}{\sum_i d_i} \tag{1}$$

Next, we calculated the Euclidean distance between each fixation i and the centroid (x_c, y_c).

$$D_i = \sqrt{(x_i - x_c)^2 + (y_i - y_c)^2} \tag{2}$$

In the last step, gaze dispersion was calculated as the square root of the duration-weighted average of squared Euclidean distance D_i^2, where D_i is the distance between fixation i and the centroid, and d_i is

the duration of fixation i.

$$\text{RMS} = \sqrt{\frac{\sum_i d_i D_i^2}{\sum_i d_i}} \quad (3)$$

6.2 Conversion of Pupil Diameter to Millimetres

The raw pupil diameter values were recorded in arbitrary units (AU), defined as the number of pixels on the eye-tracking camera corresponding to the pupil size. To convert these to millimetres, we measured an artificial eye with a known, fixed pupil size (8 mm) to obtain the corresponding number of pixels (2494 pixels). This allowed us to compute a scaling factor:

$$\text{Scaling factor} = \frac{8\,\text{mm}}{\sqrt{2494}} = 0.160 \quad (4)$$

This factor was then applied to the data using the following transformation:

$$\text{Pupil diameter (mm)} = \text{Scaling factor} \times \sqrt{\text{Pupil diameter (AU)}} \quad (5)$$

6.3 Index of Pupillary Activity (IPA)

To compute the index of pupillary activity (IPA), we segmented the pupil diameter time series of each driving block into 1-minute epochs with $50\ \%$ overlap, and downsampled the data from 1000 Hz to 250 Hz, applying zero-phase low-pass filtering beforehand. Pupil diameter signals were decomposed using a two-level periodic Discrete Wavelet Transform (DWT; symlet-16 from the Daubechies wavelet family; pywavelets 1.8.0). In the second step, abrupt fluctuations were detected by identifying local maxima in the wavelet modulus of high-frequency coefficients extracted from the second level of decomposition (Duchowski, 2018). To filter out noise, the wavelet modulus maxima coefficients were thresholded using universal thresholding, defined as

$$\lambda_{\text{univ}} = \hat{\sigma}\sqrt{2\log n}, \quad (6)$$

where $\hat{\sigma}$ is the standard deviation of the modulus maxima values and n is the number of data points (Duchowski, 2018). In the final step, the IPA score was computed by normalising the count of remaining peaks exceeding the threshold by the total signal duration $(t_2 - t_1)$:

$$\text{IPA} = \frac{\text{Number of abrupt fluctuations}}{\text{Signal duration (s)}} \quad (7)$$

Intermediate steps involved in the computation of the IPA are illustrated in Supplementary Figure 2.

7 Detailed Summary of the Linear Mixed-Effects Models

Supplementary Table 2

Summary of the Linear Mixed-Effects Models for Subjective Ratings Analysing Main Effects of Emotional Speech and Visuo-Spatial Workload, as Well as Their Interaction

Effects	dF1	df2	F	p	Post-Hoc Wilcoxon Signed-Rank Test
**Valence**					
Visuo-spatial Workload	1	207.34	7.19	.008**	
Emotional Speech	2	207.76	11.34	$<$ .001***	
HV – NV					$Z_{(46)} = -4.23$, $p < .001$***
Emotional Speech × Visuo-spatial Workload	2	206.49	4.40	.013*	
LV – NV × High – Low					Model CI $[0.11, 1.11]$
HV – NV × High – Low					Model CI $[-0.57, 0.39]$
LV – HV × High – Low					Model CI $[0.20, 1.20]$
**Arousal**					
Visuo-spatial Workload	1	222.35	7.24	.008**	
Emotional Speech	2	222.36	4.22	.016*	
LV – NV					$Z_{(47)} = -0.37$, $p = .711$
HV – NV					$Z_{(47)} = -1.94$, $p = .079$
Emotional Speech × Visuo-spatial Workload	2	222.00	3.57	.030*	
LV – NV × High – Low					Model CI $[-0.63,\ 0.23]$
HV – NV × High – Low					Model CI $[-0.06,\ 0.82]$
LV – HV × High – Low					Model CI $[-1.00,\ -0.13]$
**Frustration**					
Visuo-spatial Workload	1	220.55	40.57	$<$ .001***	
Emotional Speech	2	221.28	9.72	$<$ .001***	
HV – NV					$Z_{(47)} = -4.24$, $p < .001$***
Emotional Speech × Visuo-spatial Workload	2	220.85	7.15	.001**	
LV – NV × High – Low					Model CI $[-1.07,\ -0.25]$
HV – NV × High – Low					Model CI $[-0.39,\ 0.41]$
LV – HV × High – Low					Model CI $[-1.08,\ -0.27]$

Note. Model confidence intervals (CI) for significant interaction effects were estimated via bootstrapping of the linear mixed-effects model using 5,000 iterations. Directions of the experimental modulations are visualised in Figure 6. In the presence of a significant interaction, only interpretable main effects were reported. LV: low valence; NV: neutral valence; HV: high valence; LW: low visuo-spatial workload; HW: high visuo-spatial workload. Significance levels: * indicates $p < .05$, ** indicates $p < .01$, and *** indicates $p < .001$.

Supplementary Table 2 (continued)

Summary of the Linear Mixed-Effects Models for Subjective Ratings Analysing Main Effects of Emotional Speech and Visuo-Spatial Workload, as Well as Their Interaction

Effects	dF1	df2	F	p	Post-Hoc Wilcoxon Signed-Rank Test
**Effort**					
Visuo-spatial Workload	1	207.24	18.42	$< .001$***	
HW – LW					$Z_{(47)} = -4.48, p < .001$***
Emotional Speech	2	206.97	4.82	.009**	
LV – NV					$Z_{(47)} = -0.16, p = .874$
HV – NV					$Z_{(47)} = -3.50, p = .001$**
LV – HV					$Z_{(47)} = -2.75, p = .009$**
Emotional Speech × Visuo-spatial Workload	2	206.12	1.63	.199	
**Distraction**					
Visuo-spatial Workload	1	193.77	0.07	.791	
Emotional Speech	2	195.89	5.90	.003**	
LV – NV					$Z_{(44)} = -2.88, p = .012$*
HV – NV					$Z_{(44)} = -1.96, p = .050$
LV – HV					$Z_{(44)} = -2.22, p = .040$*
Emotional Speech × Visuo-spatial Workload	2	194.55	0.49	.613	

Note. Model confidence intervals (CI) for significant interaction effects were estimated via bootstrapping of the linear mixed-effects model using 5,000 iterations. Directions of the experimental modulations are visualised in Supplementary Figure 3A – B. Significance levels: * indicates $p < .05$, ** indicates $p < .01$, and *** indicates $p < .001$. LV: low valence; NV: neutral valence; HV: high valence; LW: low visuo-spatial workload; HW: high visuo-spatial workload.

Supplementary Table 3

Summary of the Linear Mixed-Effects Models for Supplementary Subjective Ratings Analysing Main Effects of Emotional Speech and Visuo-Spatial Workload, as Well as Their Interaction

Effects	dF1	df2	F	p	Post-Hoc Wilcoxon Signed-Rank Test
**Speech Valence**					
Visuo-spatial Workload	1	222.67	0.00	.970	
Emotional Speech	2	224.43	89.72	$< .001$***	
LV – NV					$Z_{(47)} = -0.65, p = .525$
HV – NV					$Z_{(47)} = -5.67, p < .001$***
LV – HV					$Z_{(47)} = -4.99, p < .001$***
Emotional Speech × Visuo-spatial Workload	2	222.09	1.28	.281	
**Speech Arousal**					
Visuo-spatial Workload	1	221.33	0.48	.490	
Emotional Speech	2	222.37	22.87	$< .001$***	
LV – NV					$Z_{(47)} = -3.71, p < .001$***
HV – NV					$Z_{(47)} = -0.42, p = .672$
LV – HV					$Z_{(47)} = -4.53, p < .001$***
Emotional Speech × Visuo-spatial Workload	2	221.30	0.48	.618	
**Content Misses (%)**					
Visuo-spatial Workload	1	222.61	0.20	.652	
Emotional Speech	2	223.49	12.23	$< .001$***	
LV – NV					$Z_{(47)} = -3.15, p = .005$**
HV – NV					$Z_{(47)} = -2.75, p = .008$**
LV – HV					$Z_{(47)} = -1.11, p = .269$
Emotional Speech × Visuo-spatial Workload	2	222.58	0.79	.457	
**Recall Accuracy (%)**					
Visuo-spatial Workload	1	226.00	0.94	.333	
Emotional Speech	2	225.95	37.18	$< .001$***	
LV – NV					$Z_{(47)} = -5.25, p < .001$***
HV – NV					$Z_{(47)} = -1.43, p = .154$
LV – HV					$Z_{(47)} = -5.53, p < .001$***
Emotional Speech × Visuo-spatial Workload	2	226.05	0.83	.438	

Note. Model confidence intervals (CI) for significant interaction effects were estimated via bootstrapping of the linear mixed-effects model using 5,000 iterations. Directions of the experimental modulations are visualised in Supplementary Figure 3C – F. Significance levels: * indicates $p < .05$, ** indicates $p < .01$, and *** indicates $p < .001$. LV: low valence; NV: neutral valence; HV: high valence; LW: low visuo-spatial workload; HW: high visuo-spatial workload.

Supplementary Table 4

Summary of the Linear Mixed-Effects Models for Driving Quality and Measures of Gaze Behaviour Analysing Main Effects of Emotional Speech and Visuo-Spatial Workload, as Well as Their Interaction

Effects	dF1	df2	F	p	Post-Hoc Wilcoxon Signed-Rank Test
**Driving Quality**					
Visuo-spatial Workload	1	215.53	263.22	$<$ .001***	
HW – LW					$Z_{(47)} = -6.03$, $p < .001$***
Emotional Speech	2	205.69	0.46	.633	
Emotional Speech × Visuo-spatial Workload	2	205.78	0.47	.625	
**Gaze Dispersion**					
Visuo-spatial Workload	1	181.51	9.95	.002**	
HW – LW					$Z_{(41)} = -2.82$, $p = .004$**
Emotional Speech	2	180.53	1.69	.188	
Emotional Speech × Visuo-spatial Workload	2	180.39	1.22	.297	
**Blink Activity**					
Visuo-spatial Workload	1	179.92	0.37	.543	
Emotional Speech	2	180.12	4.53	.012*	
LV – NV					$Z_{(41)} = -0.21$, $p = .843$
HV – NV					$Z_{(41)} = -1.31$, $p = .587$
LV – HV					$Z_{(41)} = -0.53$, $p = .843$
Emotional Speech × Visuo-spatial Workload	2	179.84	0.50	.610	
**Pupil Dilation**					
Visuo-spatial Workload	1	182.88	37.53	$<$ .001***	
HW – LW					$Z_{(41)} = -4.67$, $p < .001$***
Emotional Speech	2	179.35	3.95	.021*	
LV – NV					$Z_{(41)} = -1.21$, $p = .349$
HV – NV					$Z_{(41)} = -1.44$, $p = .349$
LV – HV					$Z_{(41)} = -0.32$, $p = .757$
Emotional Speech × Visuo-spatial Workload	2	180.96	0.16	.854	
**Index of Pupillary Activity (IPA)**					
Visuo-spatial Workload	1	183.76	0.42	.516	
Emotional Speech	2	184.55	0.74	.479	
Emotional Speech × Visuo-spatial Workload	2	184.78	1.74	.178	

Note. Driving quality is an aggregated score, computed as the average of the root mean square of successive differences in acceleration, steering wheel angle, mean brake actuation, mean deviation from the lane centre, and the number of traffic rule violations (Supplementary Figure 4F – J). We observed significant main effects of visuo-spatial workload in all subvariables of the aggregated score (acceleration RMSSD: $F_{(1,\ 205.00)} = 54.93$, $p < .001$; steering wheel angle RMSSD: $F_{(1,\ 210.35)} = 103.26$, $p < .001$; mean brake actuation: $F_{(1,\ 204.12)} = 106.27$, $p < .001$; mean deviation from lane centre: $F_{(1,\ 206.44)} = 25.14$, $p < .001$; number of traffic rule violations: $F_{(1,\ 230.42)} = 276.45$, $p < .001$), but no main effects of emotional speech or interaction effects. This validates their aggregation. Directions of the experimental modulations are visualised in Figure 4C – F, and Supplementary Figure 4A – E. Significance levels: * indicates $p < .05$, ** indicates $p < .01$, and *** indicates $p < .001$. LV: low valence; NV: neutral valence; HV: high valence; LW: low visuo-spatial workload; HW: high visuo-spatial workload.

8 Aperiodic Component Localised in Source Space With DICS

Modulations in components of the $1/f$-like aperiodic part of the power spectrum have recently gained attention, and their links to cognitive processes have been investigated. For instance, changes in the aperiodic exponent have been proposed to be involved in the synaptic balance between excitation and inhibition, with a more negative (steeper) slope parameter β, indicating increased inhibition (Lu et al., 2024). Additionally, aperiodic broadband power was found to decrease across the brain with increasing cognitive demand (Lu et al., 2024). Zhang et al. (2023) proposed a positive relationship between aperiodic components and cognitive control involved in the stable allocation of resources, as opposed to flexible resource allocation. It is further associated with neurophysiological changes across the lifespan (Thuwal et al., 2021), arousal states, and auditory stimulus processing (Jacob et al., 2021).

Based on these prior findings, we explored the effects of emotional speech and visuo-spatial workload on the aperiodic offset, slope, and broadband. An increase in aperiodic components was expected in response to internalised processing and emotional speech, and a decrease during externalised processing and high workload.

The permutation-based clustering revealed a significant main effect of emotional speech in the offset (rh: $p = .034$) and broadband (lh: $p = .024$; rh: $p = .010$; Supplementary Figure 9A,B). No significant clusters were found for the aperiodic slope component or the main effect of visuo-spatial workload.

The cluster of the aperiodic offset included vertices in the right temporo-parietal junction (TPJ). The broadband clusters were localised bilaterally in the supramarginal gyrus, TPJ, and extended into the superior and middle temporal sulcus.

Post-hoc tests showed increased aperiodic values during emotional compared to neutral speech (Supplementary Figure 9C,D). Offset values were significantly higher for both low (LV – NV: $M = 0.04$, $95~\%~CI~[0.02,~0.06]$; $Z_{(41)} = -5.36$, $p < .001$), and high valence speech (HV – NV: $M = 0.04$, $95~\%~CI~[0.02,~0.06]$; $Z_{(41)} = -5.14$, $p < .001$). Similarly, broadband values increased for low (LV – NV: $M = 0.05$, $95~\%~CI~[0.03,~0.07]$; $Z_{(41)} = -5.30$, $p < .001$), and high valence speech (HV – NV: $M = 0.07$, $95~\%~CI~[0.04,~0.10]$; $Z_{(41)} = -5.68$, $p < .001$) compared to neutral speech.

9 Modulations in the Non-Corrected Band Source Power Localised With DICS

When the aperiodic component is not separated from the power spectrum, permutation-based clustering reveals a main effect of emotional speech that closely resembles the effect observed in the aperiodic component analysis. Significant differences between emotional speech conditions were found in alpha- (left: $p = .007$; right: $p = .001$), beta- (left: $p < .001$; right: $p < .001$), and gamma-band power (right: $p = .03$). Similar to the patterns observed in the aperiodic offset and broadband effects (Supplementary Analysis 8), the clusters included the SMG, TPJ, and the posterior superior temporal sulcus (STS), with stronger effects observed in the right hemisphere. As with the aperiodic offset and broadband effects, post-hoc tests of average source power across significant clusters showed increased alpha (LV – NV: $M = 0.07$, $95\ \%\ CI\ [0.04,\ 0.11]$; $Z_{(41)} = -5.32$, $p < .001$; HV – NV: $M = 0.08$, $95\ \%\ CI\ [0.04,\ 0.11]$; $Z_{(41)} = -5.37$, $p < .001$), and beta power (LV – NV: $M = 0.07$, $95\ \%\ CI\ [0.04,\ 0.10]$; $Z_{(41)} = -5.30$, $p < .001$; HV – NV: $M = 0.07$, $95\ \%\ CI\ [0.04,\ 0.10]$; $Z_{(41)} = -5.34$, $p < .001$) during both negative and positive speech compared to neutral speech.

In the gamma band, power in the posterior STS and middle temporal sulcus increased for positive relative to neutral speech (HV – NV: $M = 0.07$, $95\ \%\ CI\ [0.03,\ 0.10]$; $Z_{(41)} = -4.93$, $p < .001$), and negative relative to positive speech (LV – HV: $M = -0.05$, $95\ \%\ CI\ [-0.09,\ -0.01]$; $Z_{(41)} = -2.67$, $p = .011$).

The close similarity to the aperiodic effects suggests that, without separating the $1/f$ component, changes in aperiodic activity may obscure oscillatory modulations in the frequency peak regions (above the continuous, aperiodic component).

10 Control Correlation Analysis on MEG Resting-State Data

To examine whether the antagonistic relationship between oscillatory band modulations during emotional speech and high visuo-spatial workload was specific to the experimental manipulation, we conducted a control analysis using Spearman rank correlations (r_s) on resting-state MEG data collected at the beginning of the experiment. The data were preprocessed using the same pipeline as the experimental MEG data, segmented into non-overlapping 5-second epochs, and source band power was estimated using MNE (SNR = 1) with the multitaper method (2-Hz bandwidth). Correlations were then computed within participants between band power averaged across the vertices of the main-effect clusters for socio-emotional speech and visuo-spatial workload identified via the DICS permutation-based clustering.

The results showed that the antagonistic relationship between oscillatory band modulations was specific to the experimental manipulation. In the resting-state data, gamma- and beta-band power were positively correlated ($r_s = .52$, $p = .001$, *df* $= 33$) in the cluster vertices associated with the main effect of visuo-spatial workload. For the main effect of emotional speech, we also observed positive correlations across all oscillatory band combinations: alpha-beta ($r_s = .68$, $p < .001$, *df* $= 33$), alpha-gamma ($r_s = .44$, $p = .008$, *df* $= 33$), and gamma-beta ($r_s = .56$, $p < .001$, *df* $= 33$).

11 Analyses of Decoding Performance on Personality, Demographics, Subjective Experience, and the Index of Pupillary Activity

11.1 Analyses of Inter-Individual Decoding Performance on Personality and Demographics

As meta-analytic evidence indicates that individual differences strongly influence emotion regulation (Morawetz & Basten, 2024), we further explored how low and high decoding performance in individuals was associated with personality and demographic characteristics.

To investigate the relationship between decoding performance and inter-individual differences, we divided the subjects into above-median (> 0.655) and below-median groups (≤ 0.655) based on decoding performance. The high-performing group had a mean *F*1 score of $M = 0.805$ ($95\ \%\ CI$: $[0.771, 0.840]$; $n = 20$), while the low-performing group had a mean of $M = 0.539$ ($95\ \%\ CI$: $[0.490, 0.583]$;

$n = 21$). Supplementary Figure 12A shows the mean $F1$ score and its CI across all participants (middle), as well as across those in the above-median (left), and below-median performance group (right; see Supplementary Figure 11 for the individual test decoding performances).

The median-split analysis examined how the decoding performance groups differed in their psychophysiological traits, driving experience, and demographic characteristics. The analysis revealed only a significant difference in gender proportions across decoding groups (Fisher's exact test: Odds Ratio = 0.21; $p < .029$), with fewer female participants in the high decoding group (high decoding group: $n_{female} = 6$ and $n_{male} = 14$; low decoding group: $n_{female} = 14$ and $n_{male} = 7$).

Together, these results indicate that more female participants revealed below-median decoding performance when classifying the four valence-workload conditions. This observation may be indicative of potential gender-specific differences in the integration of emotional and cognitive processes. Derntl et al. (2010) investigated gender differences in neural signatures during socio-emotional cognition and found that females and males relied on divergent processing strategies and neural networks. While females showed increased activation in subcortical limbic regions associated with emotional reactivity (signals that are not captured by MEG; Hämäläinen et al., 1993), males displayed increased activation in cortical regions associated with cognitive aspects of social cognition (Derntl et al., 2010).

11.2 Analyses of Decoding Group Membership on Subjective Measures and the Index of Pupillary Activity

In a second exploratory analysis, we examined whether decoding group membership (i.e., the ability to decode individual neural signatures correctly) influenced the co-modulation of valence and workload on subjective measures (valence, arousal, frustration, effort, and distraction) and the IPA, as hypothesised in H.3. This was tested by including decoding group membership as a three-way interaction term in the LMMs. While no effects of decoding group were found on subjective measures, a significant three-way interaction was observed for the IPA ($F_{(1,\ 92.15)} = 5.99$, $p = .016$; excluded outlier observations: $12.50\ \%$; Supplementary Figure 13). In the high decoding group, IPA showed a valence-by-workload interaction: During low visuo-spatial workload, IPA increased for negative speech and decreased for positive speech. Conversely, under high workload, IPA increased for positive speech and decreased for negative speech. In contrast, the low decoding group showed no such interaction. In this group, IPA slightly increased with workload regardless of speech valence (LMM $95\ \%\ CI$ of the interaction effect: $[0.30,\ 2.95]$; bootstrapping with 5,000 iterations).

In summary, the three-way interaction hints at a functional connection between pupillary discontinuities

and fronto-temporal mechanisms underlying emotion-cognition integration and attentional control.

12 Supplementary Figures

Supplementary Figure 1

Illustrative Time Series of Acceleration Change Rate, Excluded Muscle-Related Independent Component, as well as Raw and Cleaned Foot-Movement Related Magnetometer Channel

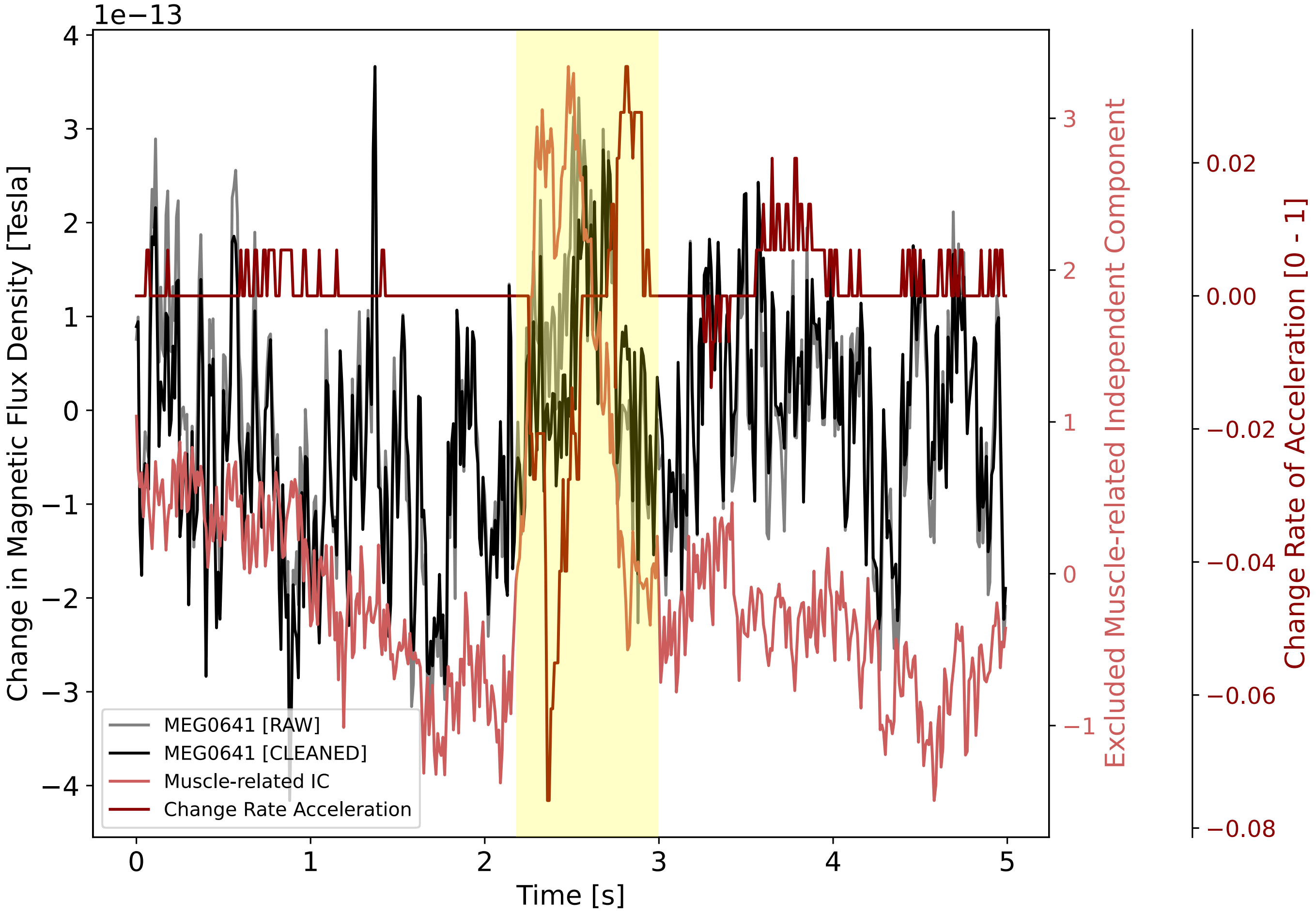

Note. Example time series showing the acceleration change rate, the excluded muscle-related independent component, and the MEG signal from magnetometer channel MEG0641 located over the right-foot representation of the left motor cortex. Acceleration values range between 0 and 1. The light-yellow shading indicates a segment that contains a muscle-activity event. Grey: raw MEG signal; black: cleaned MEG signal after independent component (IC) removal; light red: time course of the excluded independent component most strongly associated with muscle artefacts; dark red: acceleration change rate (first derivative).

Supplementary Figure 2

Visualisation of the Intermediate Steps Involved in the Computation of the Index of Pupillary Activity (IPA)

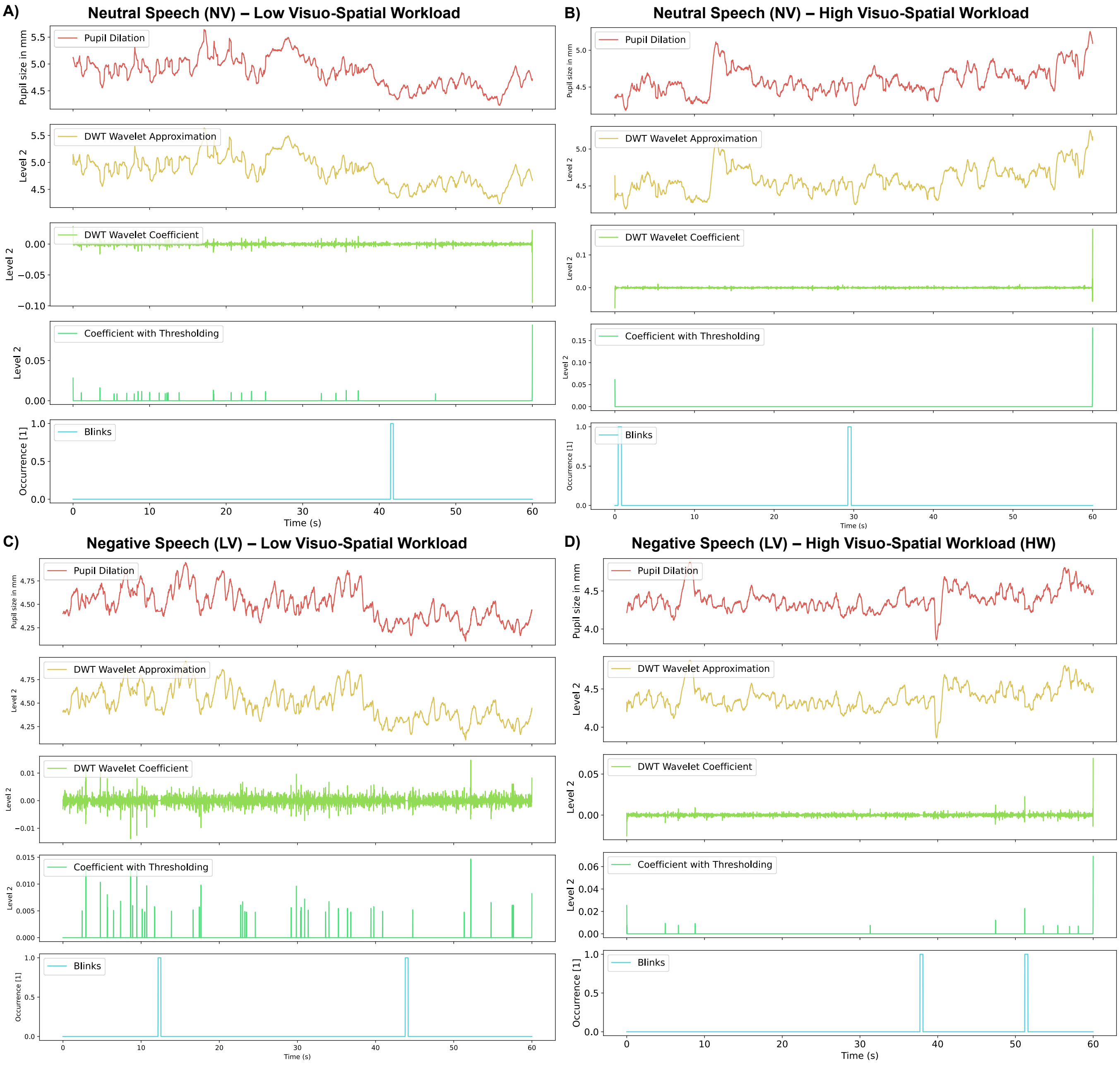

Note. The process is demonstrated using four illustrative conditions. Conditions were selected to represent the most pronounced differences in auditory complexity/arousal and visuo-spatial workload. Pupil dilation (top row) and blink occurrence (bottom row) are visualised alongside the wavelet approximation and coefficients. DWT: Discrete Wavelet Transform.

Supplementary Figure 3

Descriptive Statistics for Subjective Ratings (Excluding Ratings With a Significant Interaction Effect)

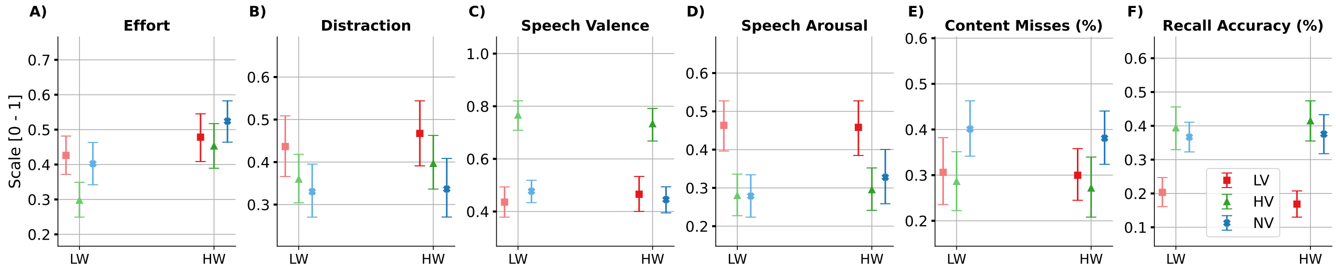

Note. Coloured symbols and error bars represent the bootstrapped grand averages for each condition, along with their Bonferroni-corrected 2.5th and 97.5th percentile confidence intervals (CI) across participants. LV: low valence; NV: neutral valence; LW: low visuo-spatial workload; HW: high visuo-spatial workload.

Supplementary Figure 4

Descriptive Statistics for Correlates of Gaze and Driving Behaviour

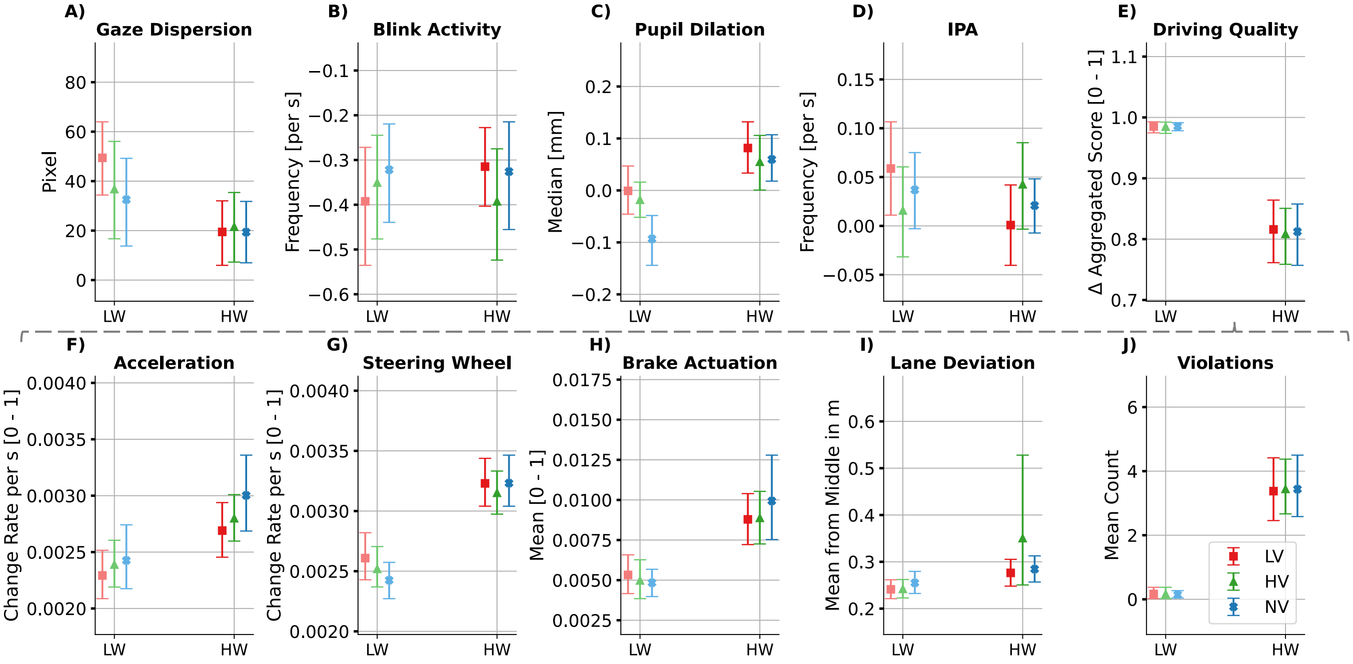

Note. Coloured symbols and error bars represent the bootstrapped grand averages for each condition, along with their Bonferroni-corrected 2.5^{th} and 97.5^{th} percentile confidence intervals (CI) across participants. LV: low valence; NV: neutral valence; HV: high valence; LW: low visuo-spatial workload; HW: high visuo-spatial workload.

Supplementary Figure 5

Frequency Spectra of Source-Localised Oscillatory Power Obtained Using Dynamic Imaging of Coherent Sources (DICS)

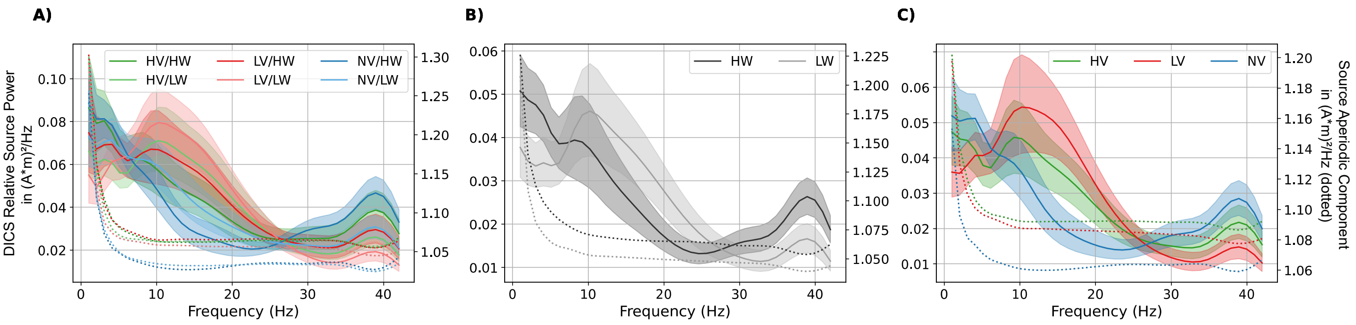

Note. Panels (A) to (C) show the oscillatory source power when subtracting the aperiodic component (solid lines) and estimated aperiodic component (dotted lines). Spectra correspond to the conditions of (A) single effects, (B) main effect of visuo-spatial workload, and (C) main effects of emotional speech. LV: low valence; NV: neutral valence; HV: high valence; LW: low visuo-spatial workload; HW: high visuo-spatial workload.

Supplementary Figure 6

Oscillatory Source Power After Subtraction of the Aperiodic Component per Frequency Band in the Main Effect Conditions

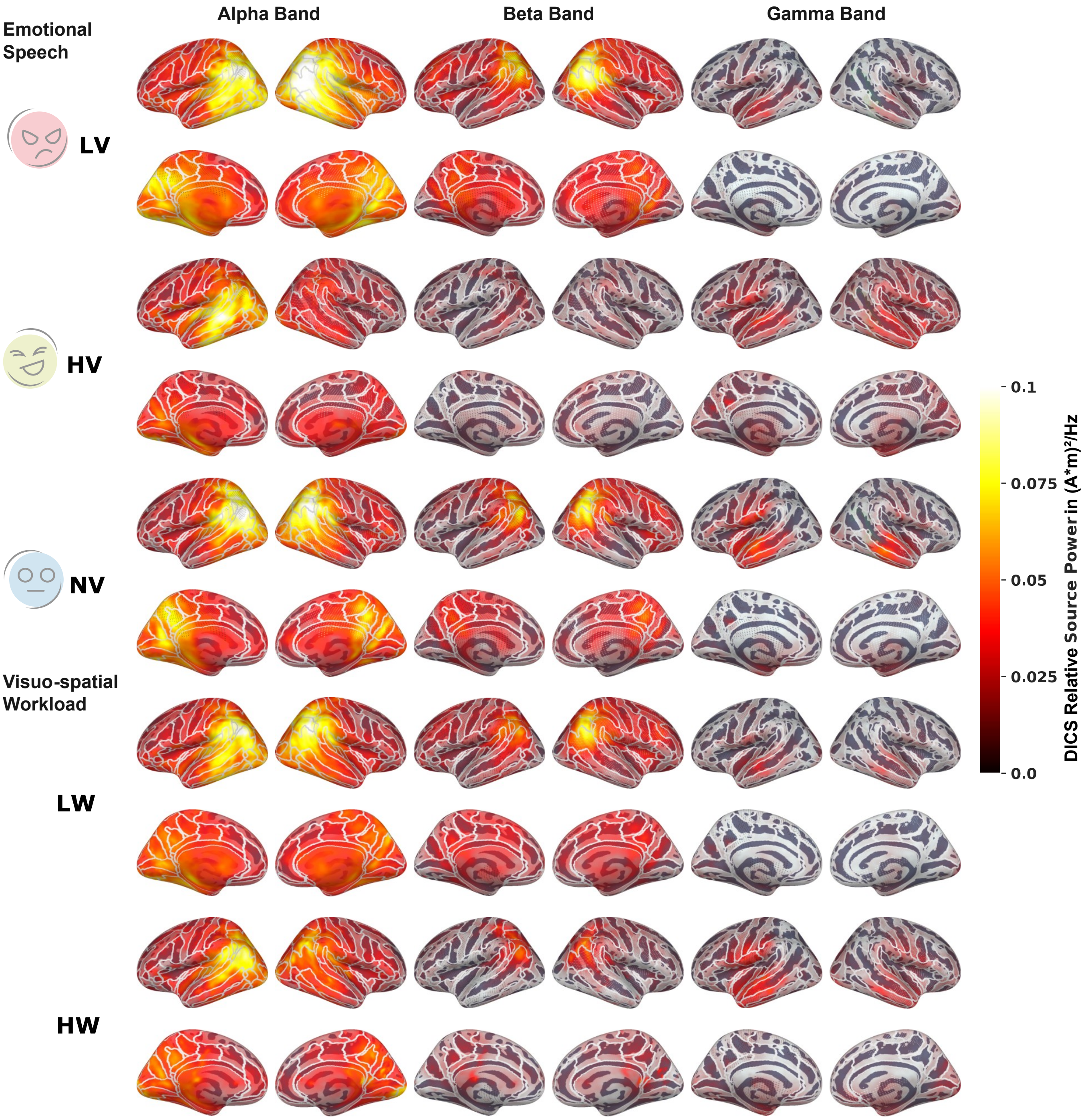

Note. Source power was estimated using dynamic imaging of coherent sources (DICS). LV: low valence; NV: neutral valence; HV: high valence; LW: low visuo-spatial workload; HW: high visuo-spatial workload.

Supplementary Figure 7

Oscillatory Source Power Without Subtraction of the Aperiodic Component per Frequency Band in the Main Effect Conditions

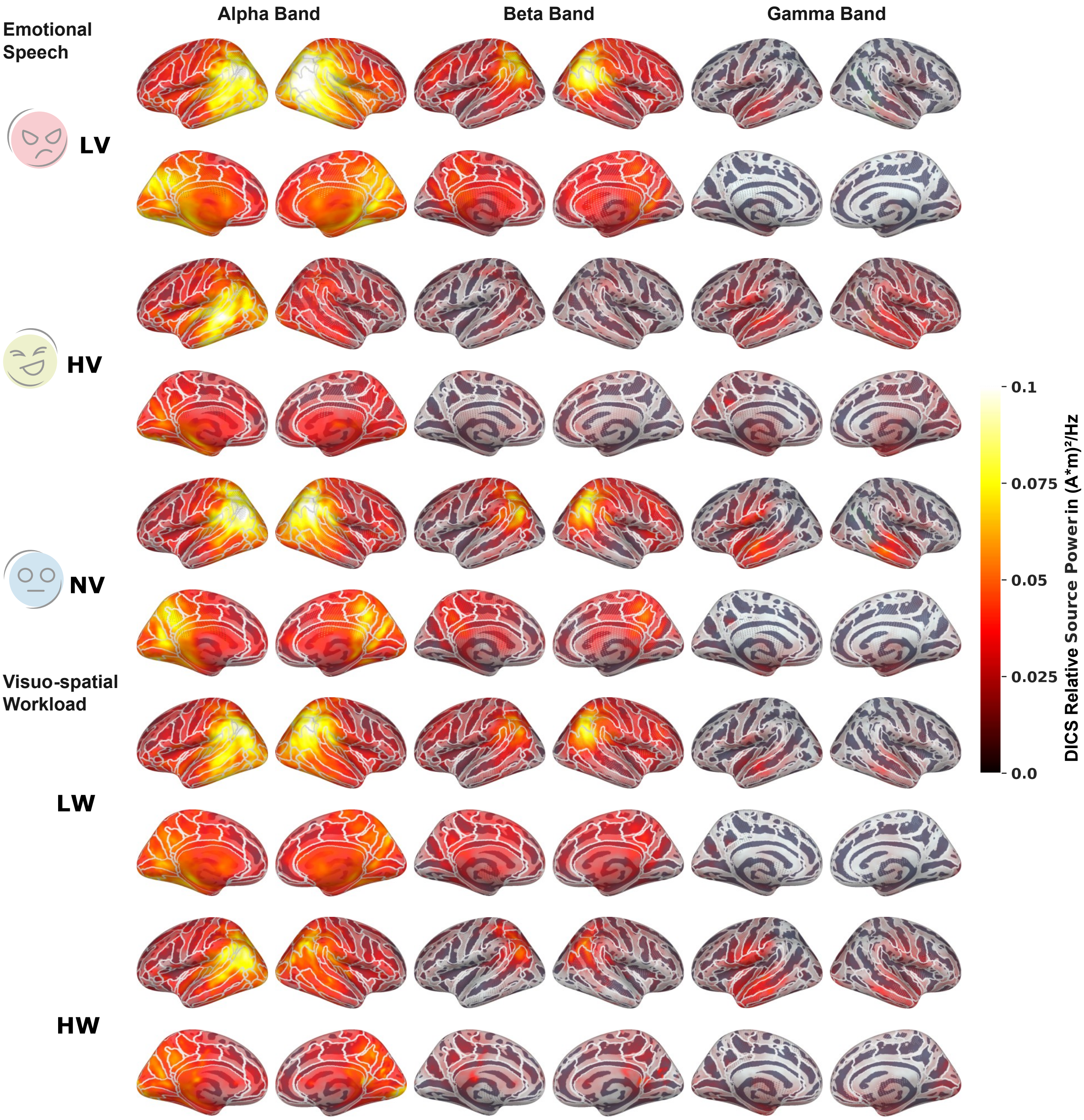

Note. Source power was estimated using dynamic imaging of coherent sources (DICS). LV: low valence; NV: neutral valence; HV: high valence; LW: low visuo-spatial workload; HW: high visuo-spatial workload.

Supplementary Figure 8

Descriptive Statistics for Source Power in Frequency Bands Within Significant Clusters

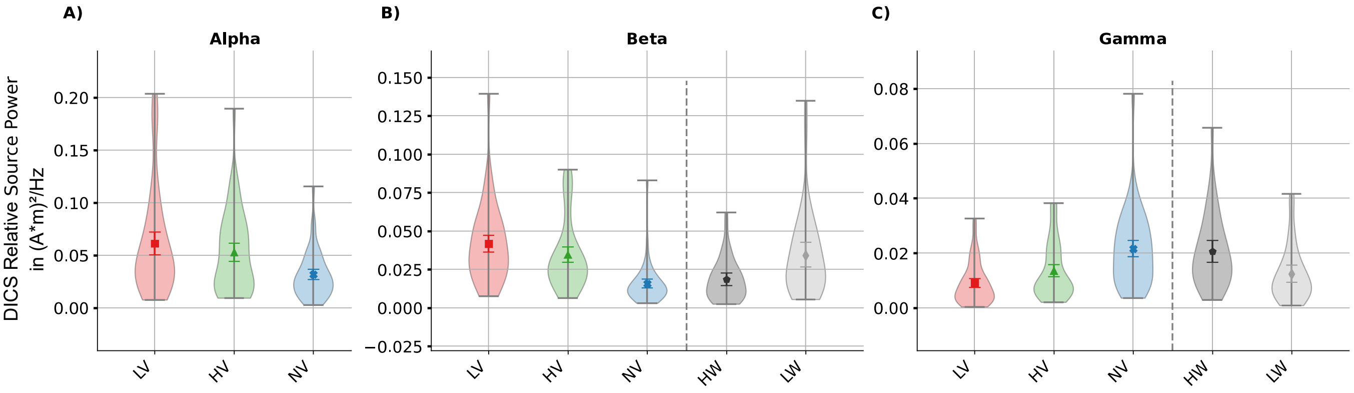

Note. Source power was estimated using dynamic imaging of coherent sources (DICS). Coloured dots and error bars represent the bootstrapped grand averages for each condition, along with their Bonferroni-corrected 2.5^{th} and 97.5^{th} percentile confidence intervals (CI) across participants. The shaded area indicates the estimated probability density of the data. LV: low valence; NV: neutral valence; HV: high valence; LW: low visuo-spatial workload; HW: high visuo-spatial workload.

Supplementary Figure 9

Permutation-Based Spatial Clustering Results of the Main Effect Emotional Speech on the Aperiodic Components

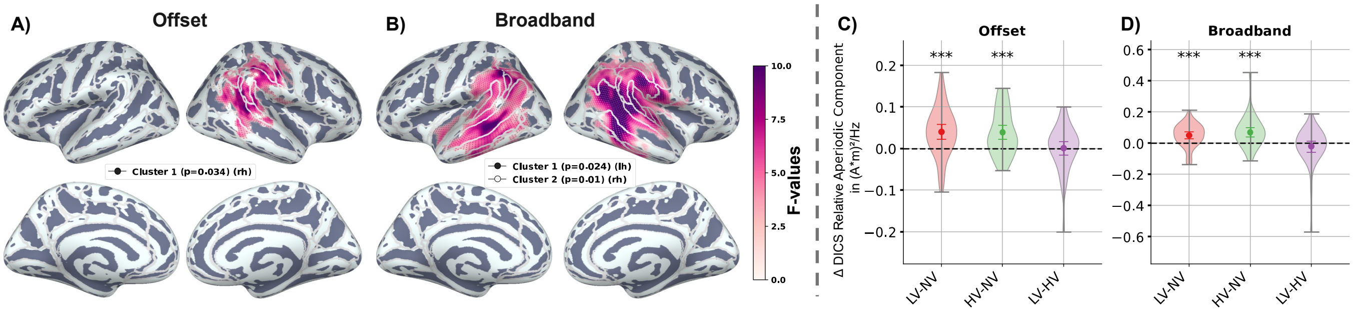

Note. Spatial distribution of significant F-values from the F-test clusters for the main effect of emotional speech is shown in Panel (A) for the offset and (B) for the broadband. Source power was estimated using dynamic imaging of coherent sources (DICS). Panels (C) and (D) show the contrasts of the post-hoc comparisons conducted using Wilcoxon signed-rank tests (FDR-corrected). Contrasts represent the difference between the conditions in the averaged aperiodic component values from significant vertices. Coloured dots and error bars represent the bootstrapped grand averages for each contrast, along with their Bonferroni-corrected 2.5^{th} and 97.5^{th} percentile confidence intervals (CI) across participants. The shaded area indicates the estimated probability density of the data. Significance levels: * indicates $p < .05$, ** indicates $p < .01$, and *** indicates $p < .001$. LV: low valence; NV: neutral valence; HV: high valence; LW: low visuo-spatial workload; HW: high visuo-spatial workload.

Supplementary Figure 10

Permutation-Based Spatial Clustering Results of the Main Effect Emotional Speech Performed on the Source Power Without Subtracting the Aperiodic Component

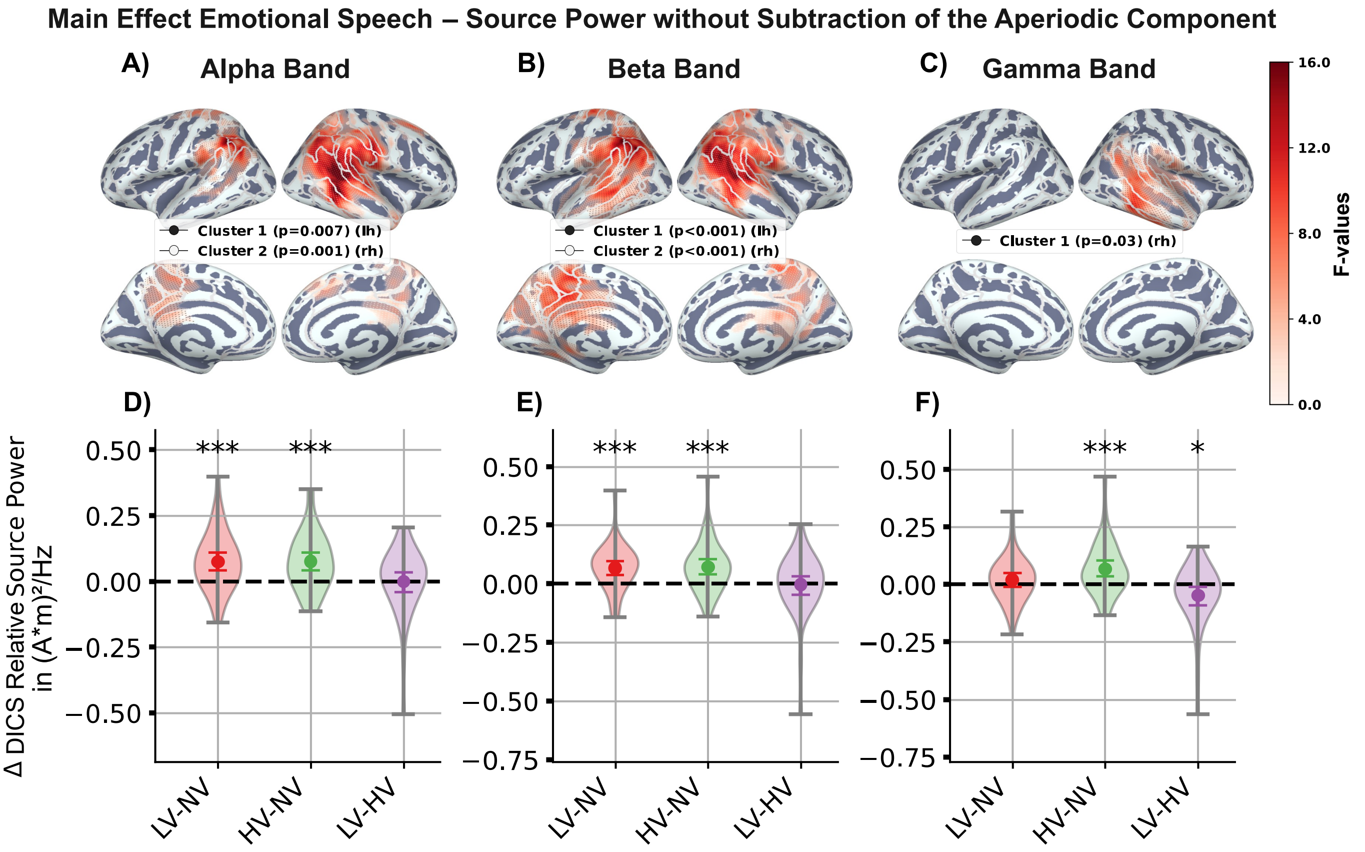

Note. Spatial distribution of significant F-values from the F-test clusters for the main effect of emotional speech on (A) alpha-, (B) beta-, and (C) gamma-band power. Source power was estimated using dynamic imaging of coherent sources (DICS). Panels (D) to (F) show the contrasts of the post-hoc comparisons conducted using Wilcoxon signed-rank tests (FDR-corrected). Contrasts represent the difference between the conditions in the averaged band power from significant vertices. Coloured dots and error bars represent the bootstrapped grand averages for each contrast, along with their Bonferroni-corrected 2.5^{th} and 97.5^{th} percentile confidence intervals (CI) across participants. The shaded area indicates the estimated probability density of the data. Significance levels: * indicates $p < .05$, ** indicates $p < .01$, and *** indicates $p < .001$. LV: low valence; NV: neutral valence; HV: high valence; LW: low visuo-spatial workload; HW: high visuo-spatial workload.

Supplementary Figure 11

Bootstrapped Mean F1 Scores Across Folds per Subject

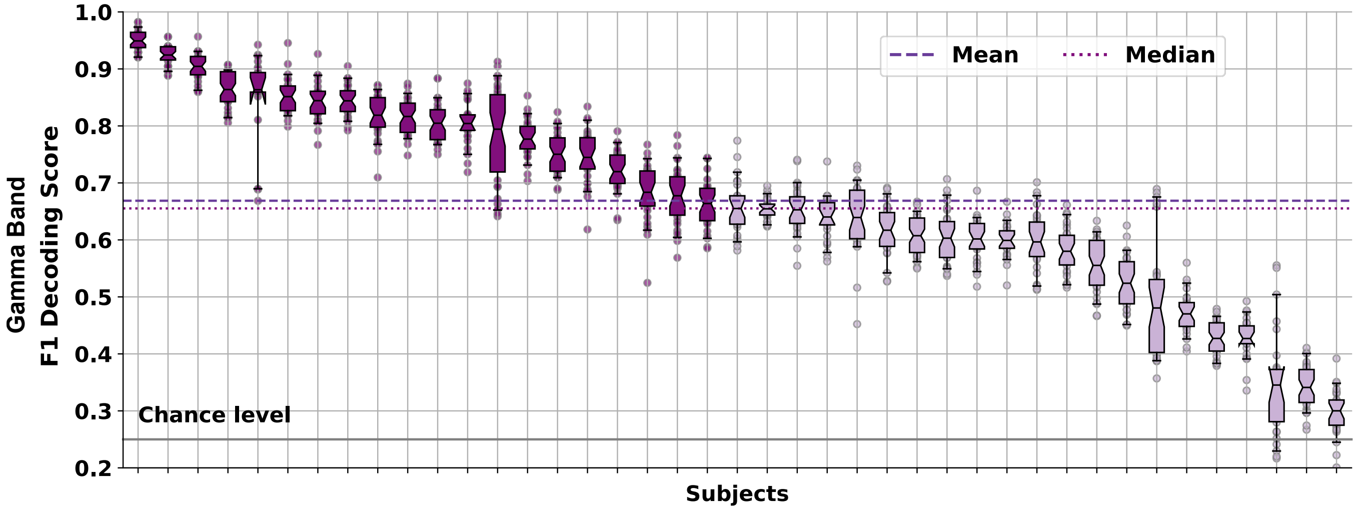

Note. Notches in the boxplots indicate the $95\ \%$ confidence interval of the bootstrapped mean. The box spans the interquartile range (25th to 75th percentile), and the whiskers represent the 5th and 95th percentiles. The purple dashed line indicates the overall mean, the purple dotted line the median, and the grey line the chance level at 0.25.

Supplementary Figure 12

Average Performance and Confusion Matrix of the Decoding

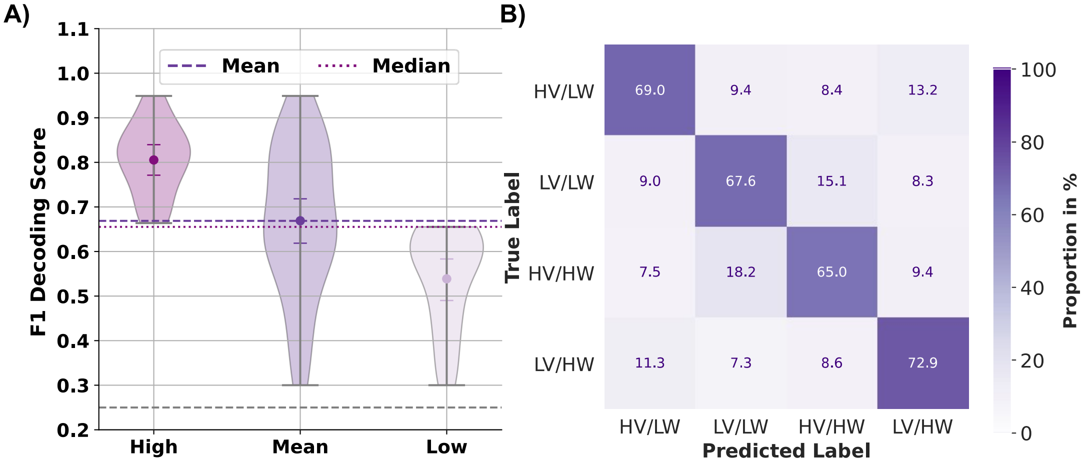

Note. (A) Mean $F1$ score across all subjects (mean; middle) and within the above median (high; left) and below median (low; right) decoding groups. Purple dashed line represents the mean, and dotted line the median. Dashed grey line represents the chance level at 0.25. Dots and error bars represent the bootstrapped grand averages, along with their Bonferroni-corrected 2.5^{th} and 97.5^{th} percentile confidence intervals (CI). (B) Confusion matrix displaying the proportion of models' predicted class label relative to the true class labels, averaged across folds and participants. LV: low valence; HV: high valence; LW: low visuo-spatial workload; HW: high visuo-spatial workload.

Supplementary Figure 13

Three-way Interaction Between Decoding Group, Valence of Speech, and Workload

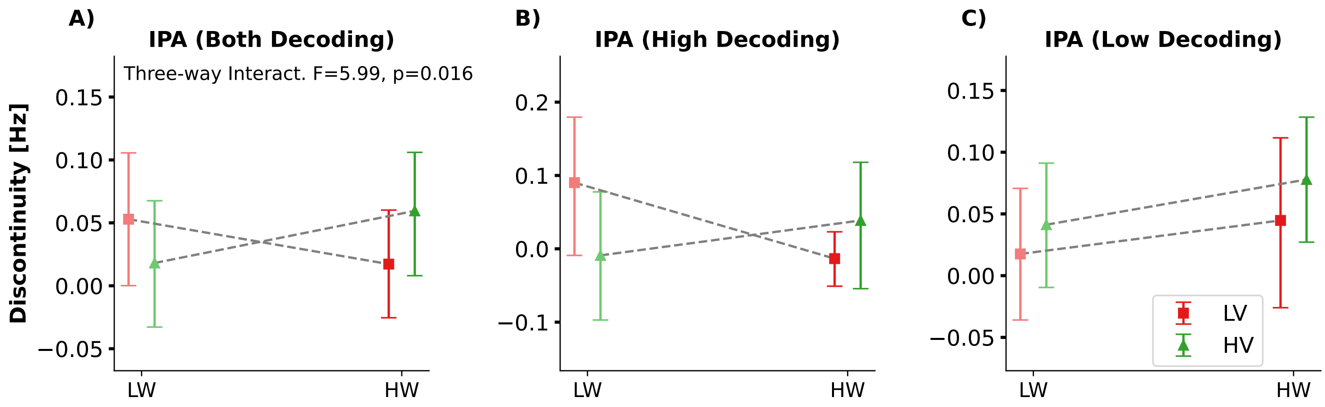

Note. Coloured dots and error bars represent the bootstrapped grand averages and their Bonferroni-corrected $95\ \%$ confidence intervals per condition for the (A) full sample, (B) high decoding group (above median), and (C) low decoding group (below median). LV: low valence; HV: high valence; LW: low visuo-spatial workload; HW: high visuo-spatial workload.

Supplementary Figure 14

Decoding Patterns of the Four Common Spatial Pattern Components for Four Exemplary Subjects

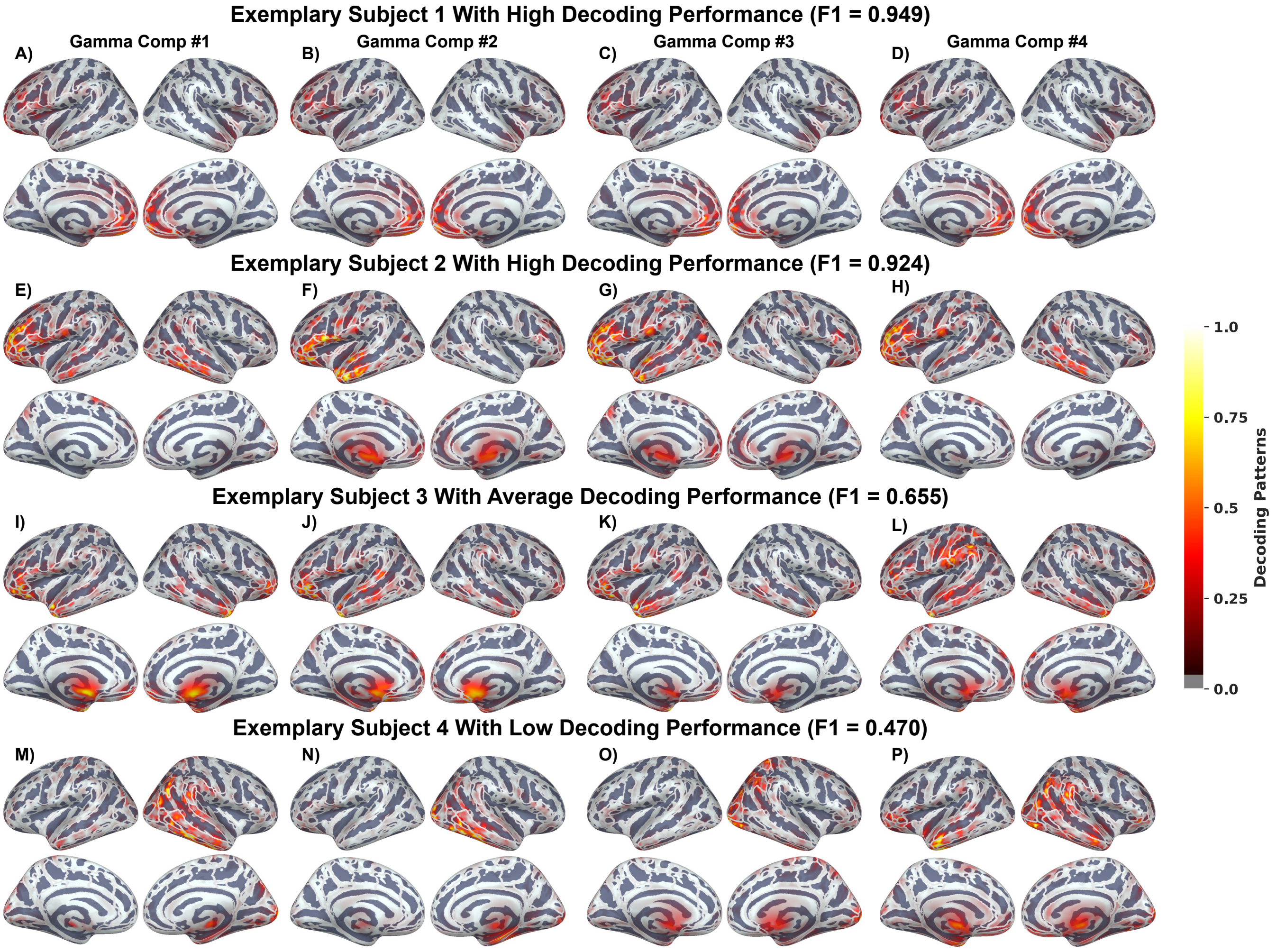

Note. Components used in the linear discriminant analysis (LDA) are visualised with normalised values (range: 0-1) in the lateral (upper row) and medial view (lower row). Panels (A) to (H) show two exemplary subjects with high decoding performance ($F1 = 0.949$ and $F1 = 0.924$), panels (I) to (L) show one exemplary subject with average decoding performance ($F1 = 0.655$), and panels (M) to (P) depict an exemplary subject with low decoding performance ($F1 = 0.470$). In subjects with high decoding performance, patterns were largely restricted to fronto-temporal vertices. In contrast, subjects with lower decoding performance displayed more widespread activation patterns extending into parietal, occipital, and inferior temporal regions. These distinct patterns could point to differences in the regulatory and processing strategies engaged during socio-emotional speech under high visuo-spatial workload (i.e., more focal frontotemporal regulation versus more widespread, potentially compensatory visual recruitment).

Supplementary Figure 15

Average Gamma Source Power Across Vertices With Power Exceeding the 90th Percentile From the Decoding Components of the Common Spatial Pattern Algorithm

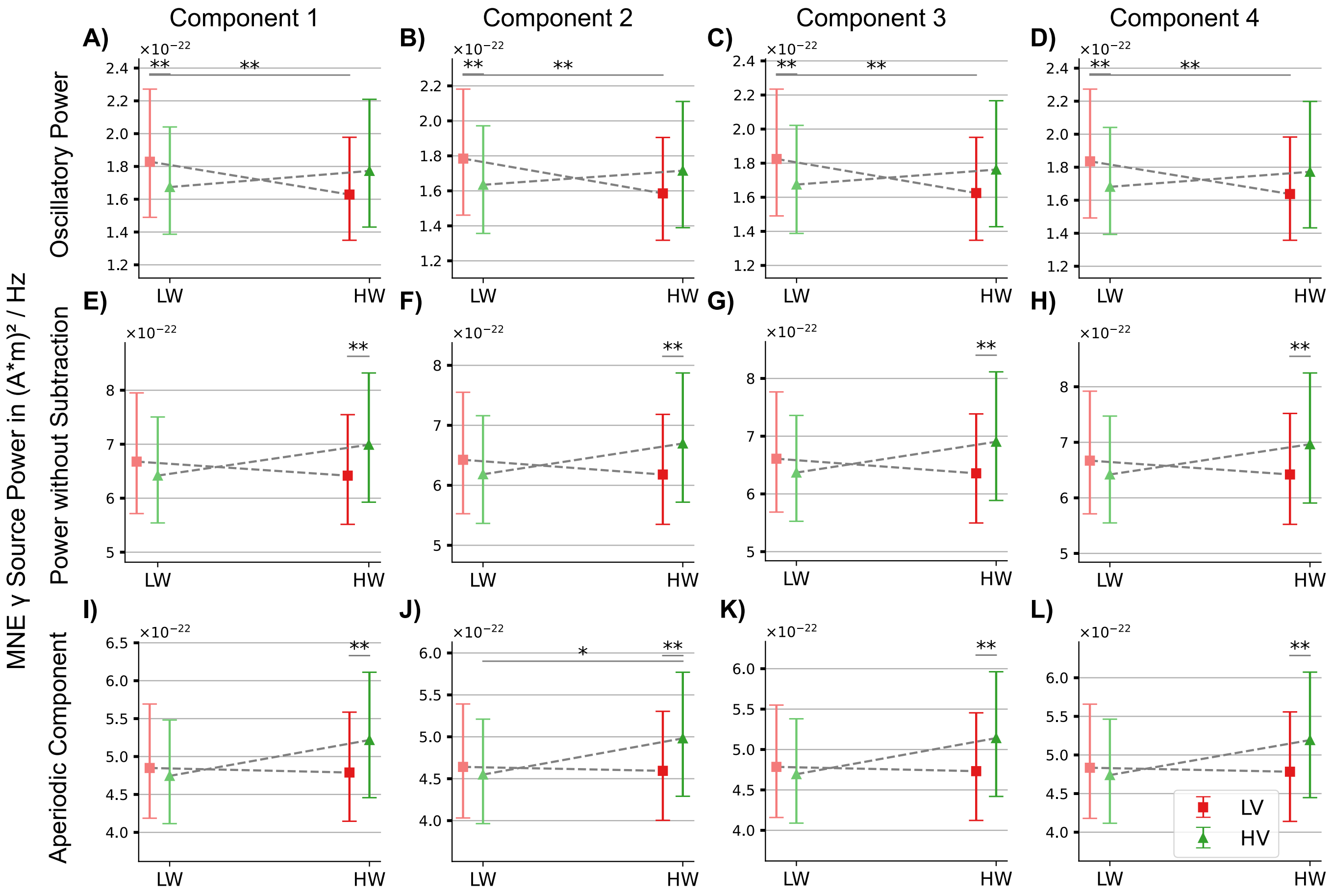

Note. The CSP components were used in the four-class classification of the interaction. Panels (A) – (D) show the gamma source power localised with MNE and corrected with the aperiodic component. Panels (E) – (H) show the gamma source power without separation of the aperiodic component. Panels (I) – (L) show the aperiodic broadband. Post-hoc comparisons were conducted using Wilcoxon signed-rank tests (FDR-corrected). Coloured symbols and error bars represent the bootstrapped grand averages for each condition, along with their Bonferroni-corrected 2.5th and 97.5th percentile confidence intervals (CI) across participants. Significance levels from the Wilcoxon signed-rank test: *** for $p < .001$, ** for $p < .01$, * for $p < .05$. LV: low valence; HV: high valence; LW: low visuo-spatial workload; HW: high visuo-spatial workload.

Supplementary Figure 16

Permutation-Based Spatial Clustering Results of the Gamma-Band Interaction Effect and Source Estimates

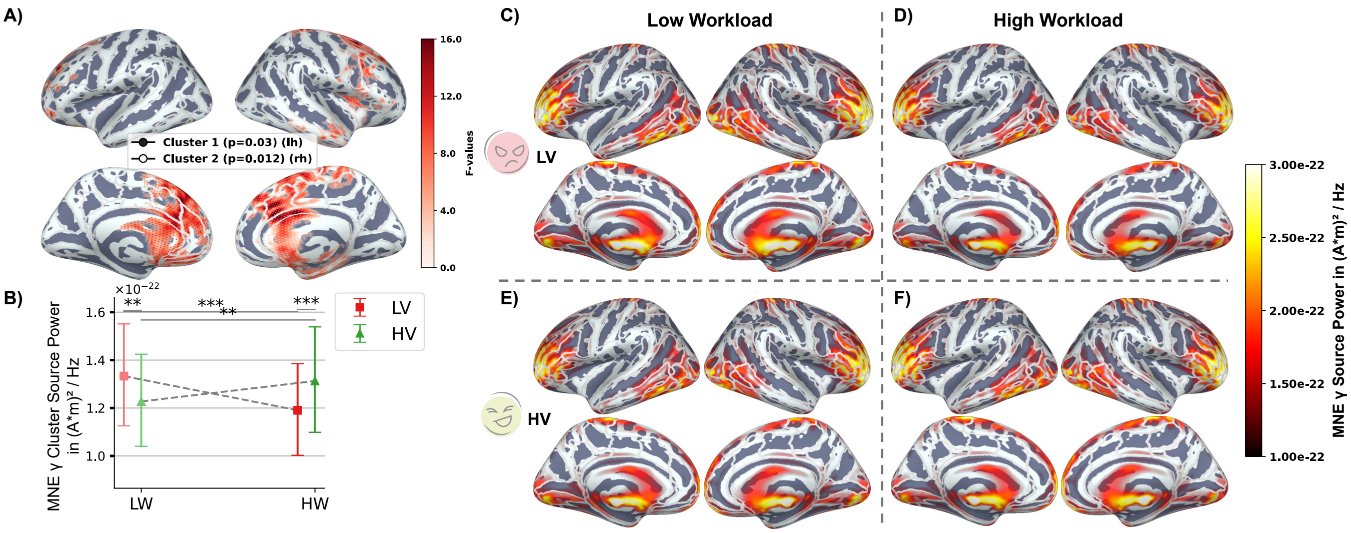

Note. Spatial distribution of significant *F*-values of the clustering is visualised in Panel (A). Clustering was performed on oscillatory gamma-band power after subtracting the aperiodic component. Source power was estimated using the minimum norm estimation (MNE) method. Panel (B) shows the gamma-band source power within significant vertices for the conditions. Post-hoc comparisons were conducted using Wilcoxon signed-rank tests (FDR-corrected). Coloured symbols and error bars represent the bootstrapped grand averages for each condition, along with their Bonferroni-corrected 2.5^{th} and 97.5^{th} percentile confidence intervals (CI) across participants. Panels (C) to (F) display the source gamma-band power per condition. Significance levels from the Wilcoxon signed-rank test: *** for $p < .001$, ** for $p < .01$, * for $p < .05$. LV: low valence; HV: high valence; LW: low visuo-spatial workload; HW: high visuo-spatial workload.

Supplementary Figure 17

Permutation-Based Spatial Clustering Results of the Interaction Effect on the Aperiodic Broadband Component

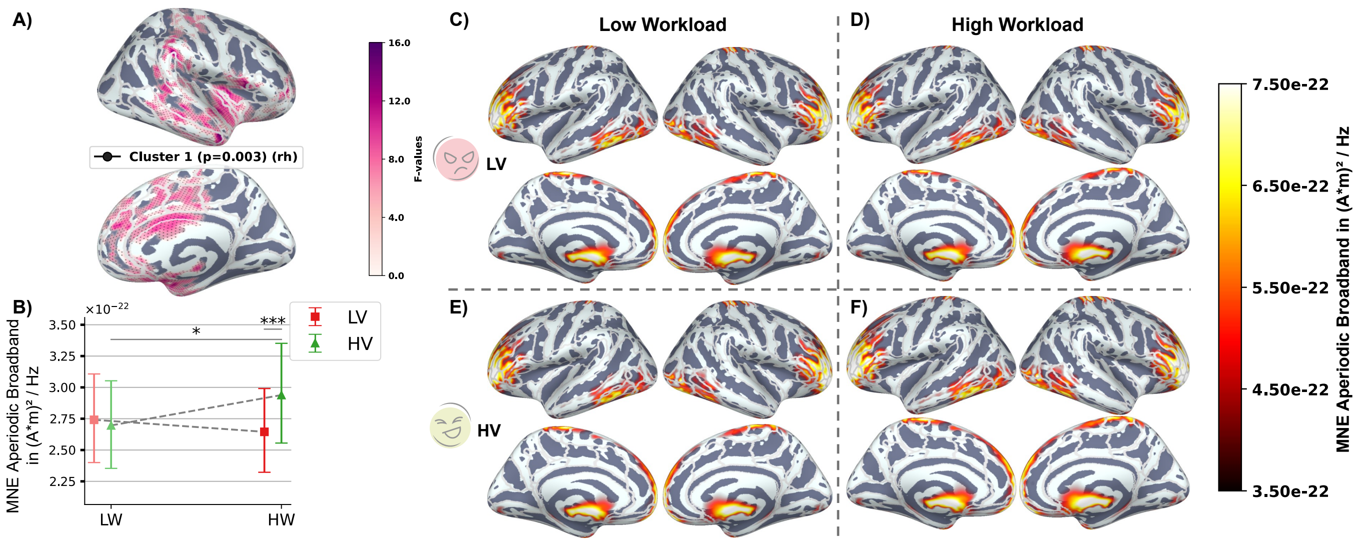

Note. Panel (A) shows the spatial distribution of significant *F*-values of the clustering using the aperiodic broadband. Source power was estimated using the minimum norm estimation (MNE) method. Panel (B) shows the averaged aperiodic broadband per condition, averaged across significant vertices. Post-hoc comparisons were conducted using Wilcoxon signed-rank tests (FDR-corrected). Coloured symbols and error bars represent the bootstrapped grand averages for each condition, along with their Bonferroni-corrected 2.5^{th} and 97.5^{th} percentile confidence intervals (CI) across participants. Panels (C) to (F) display the aperiodic broadband component per condition in source space. Significance levels from the Wilcoxon signed-rank test: *** for $p < .001$, ** for $p < .01$, * for $p < .05$. LV: low valence; HV: high valence; LW: low visuo-spatial workload; HW: high visuo-spatial workload.

Supplementary Figure 18

Permutation-Based Spatial Clustering Results of the Gamma-Band Interaction Effect Without Subtracting the Aperiodic Component

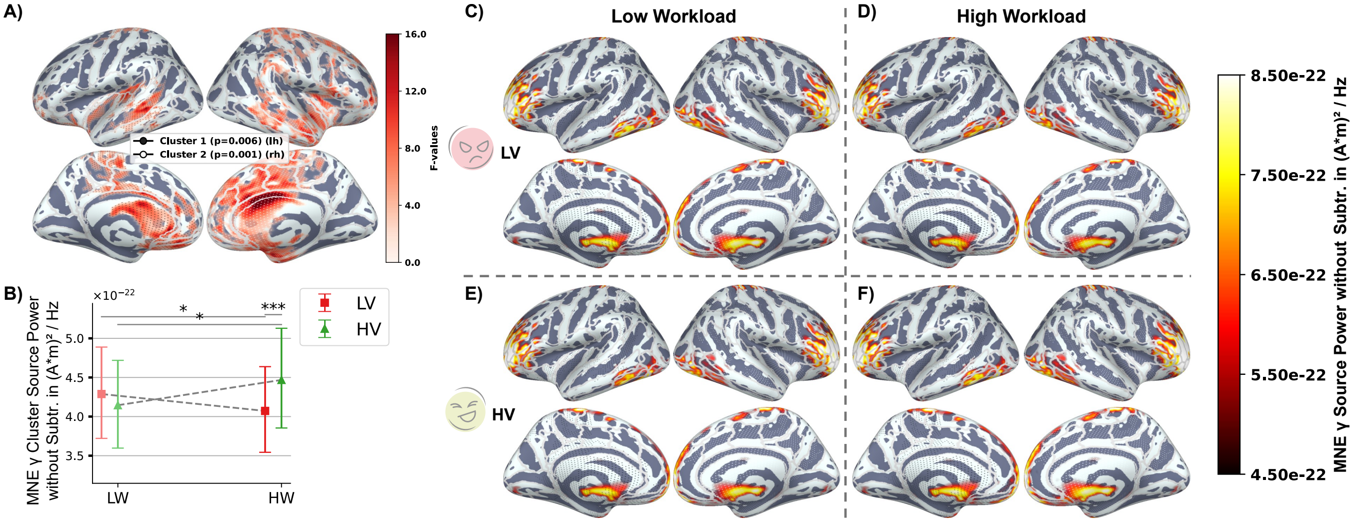

Note. Spatial distribution of significant *F*-values of the clustering is visualised in Panel (A). Clustering was performed on oscillatory gamma-band power without subtracting the aperiodic component. Source power was estimated using the minimum norm estimation (MNE) method. Panel (B) shows the gamma-band source power within significant vertices for the conditions. Post-hoc comparisons were conducted using Wilcoxon signed-rank tests (FDR-corrected). Coloured symbols and error bars represent the bootstrapped grand averages for each condition, along with their Bonferroni-corrected 2.5^{th} and 97.5^{th} percentile confidence intervals (CI) across participants. Panels (C) to (F) display the source gamma-band power per condition. Significance levels from the Wilcoxon signed-rank test: *** for $p < .001$, ** for $p < .01$, * for $p < .05$. LV: low valence; HV: high valence; LW: low visuo-spatial workload; HW: high visuo-spatial workload.

References

Blankertz, B., Tomioka, R., Lemm, S., Kawanabe, M., & Müller, K.-R. (2008). Optimizing spatial filters for robust EEG single-trial analysis. *IEEE Signal Processing Magazine*, *25*(1), 41–56. https://doi.org/10.1109/MSP.2008.4408441

Derntl, B., Finkelmeyer, A., Eickhoff, S., Kellermann, T., Falkenberg, D. I., Schneider, F., & Habel, U. (2010). Multidimensional assessment of empathic abilities: Neural correlates and gender differences. *Psychoneuroendocrinology*, *35*(1), 67–82. https://doi.org/10.1016/j.psyneuen.2009.10.006

Donoghue, T., Haller, M., Peterson, E. J., Varma, P., Sebastian, P., Gao, R., Noto, T., Lara, A. H., Wallis, J. D., Knight, R. T., Shestyuk, A., & Voytek, B. (2020). Parameterizing neural power spectra into periodic and aperiodic components. *Nature Neuroscience*, *23*(12), 1655–1665. https://doi.org/10.1038/s41593-020-00744-x

Duchowski, A. T. (2018). The index of pupillary activity: Measuring cognitive load vis-à-vis task difficulty with pupil oscillation. *Proceedings of the 2018 CHI Conference on Human Factors in Computing Systems*, 282:1–282:13. https://doi.org/10.1145/3173574.3173856

Grosse-Wentrup, M., & Buss, M. (2008). Multiclass common spatial patterns and information theoretic feature extraction. *IEEE Transactions on Biomedical Engineering*, *55*(8), 1991–2000. https://doi.org/10.1109/TBME.2008.921154

Hämäläinen, M. S., Hari, R., Ilmoniemi, R. J., Knuutila, J., & Lounasmaa, O. V. (1993). Magnetoencephalography – Theory, instrumentation, and applications to noninvasive studies of the working human brain. *Reviews of Modern Physics*, *65*(2), 413–497. https://doi.org/10.1103/RevModPhys.65.413

Jacob, M. S., Roach, B. J., Sargent, K. S., Mathalon, D. H., & Ford, J. M. (2021). Aperiodic measures of neural excitability are associated with anticorrelated hemodynamic networks at rest: A combined EEG-fMRI study. *NeuroImage*, *245*, 118705. https://doi.org/10.1016/j.neuroimage.2021.118705

Lingelbach, K., Vukelić, M., & Rieger, J. W. (2024). GAUDIE: Development, validation, and exploration of a naturalistic German auditory emotional database. *Behavior Research Methods*, *56*, 2049–2063. https://doi.org/10.3758/s13428-023-02135-z

Lu, R., Dermody, N., Duncan, J., & Woolgar, A. (2024). Aperiodic and oscillatory systems underpinning human domain-general cognition. *Communications Biology*, *7*, 1643. https://doi.org/10.1038/s42003-024-07397-7

Morawetz, C., & Basten, U. (2024). Neural underpinnings of individual differences in emotion regulation: A systematic review. *Neuroscience & Biobehavioral Reviews*, *162*, 105727. https://doi.org/10.1016/j.neubiorev.2024.105727

Thuwal, K., Banerjee, A., & Roy, D. (2021). Aperiodic and periodic components of ongoing oscillatory brain dynamics link distinct functional aspects of cognition across adult lifespan. *eNeuro*, *8*(5). https://doi.org/10.1523/ENEURO.0224-21.2021

Zhang, C., Stock, A.-K., Mückschel, M., Hommel, B., & Beste, C. (2023). Aperiodic neural activity reflects metacontrol. *Cerebral Cortex*, *33*(12), 7941–7951. https://doi.org/10.1093/cercor/bhad089
